# A Bayesian context fear learning algorithm/automaton

**DOI:** 10.3389/fnbeh.2015.00112

**Published:** 2015-05-27

**Authors:** Franklin B. Krasne, Jesse D. Cushman, Michael S. Fanselow

**Affiliations:** ^1^Department of Psychology, University of California Los AngelesLos Angeles, CA, USA; ^2^Brain Research Institute, University of California Los AngelesLos Angeles, CA, USA; ^3^Department of Psychiatry and Biobehavioral Sciences, University of California Los AngelesLos Angeles, CA, USA

**Keywords:** fear conditioning, hippocampus, amygdala, model, Bayesian, context, computational neuroscience

## Abstract

Contextual fear conditioning is thought to involve the synaptic plasticity-dependent establishment in hippocampus of representations of to-be-conditioned contexts which can then become associated with USs in the amygdala. A conceptual and computational model of this process is proposed in which contextual attributes are assumed to be sampled serially and randomly during contextual exposures. Given this assumption, moment-to-moment information about such attributes will often be quite different from one exposure to another and, in particular, between exposures during which representations are created, exposures during which conditioning occurs, and during recall sessions. This presents challenges to current conceptual models of hippocampal function. In order to meet these challenges, our model's hippocampus was made to operate in different modes during representation creation and recall, and non-hippocampal machinery was constructed that controlled these hippocampal modes. This machinery uses a comparison between contextual information currently observed and information associated with existing hippocampal representations of familiar contexts to compute the Bayesian Weight of Evidence that the current context is (or is not) a known one, and it uses this value to assess the appropriateness of creation or recall modes. The model predicts a number of known phenomena such as the immediate shock deficit, spurious fear conditioning to contexts that are absent but similar to actually present ones, and modulation of conditioning by pre-familiarization with contexts. It also predicts a number of as yet unknown phenomena.

## Introduction

During Pavlovian fear conditioning, animals become afraid of both specific cues that predict the imminent onset of aversive events such as foot shock, and the situation or “context” in which the shock occurred. Fear conditioning provides one of neuroscience's most promising and active arenas for analyzing neural mechanisms of learning, generally. Both cue and context fear conditioning seem to be due to plastic change at synapses within the amygdala (reviewed by Fanselow and LeDoux, 1999; Blair et al., 2001), but there are striking differences in the phenomenology and the neural circuitry of cue and context fear conditioning. Anatomically, cued fear seems to involve pathways from thalamus and cortex directly to the lateral nucleus of amygdala (LeDoux and Clugnet, 1990; LeDoux et al., 1990; Romanski and LeDoux, 1992; Boatman and Kim, 2006), whereas context fear utilizes a pathway from cortex to hippocampus to basal amygdala (Young et al., 1994; Maren and Fanselow, 1995; Bast et al., 2003; Matus-Amat et al., 2004; Calandreau et al., 2005; Parsons and Otto, 2008; Schenberg and Oliveira, 2008; Onishi and Xavier, 2010), though the exact route of this latter pathway remains under investigation (Fanselow and Dong, 2010). Moreover, development of context fear depends not only on synaptic plasticity within amygdala but also within hippocampus (Kiernan and Cranney, 1992; Fanselow, 2000; Rudy and O'Reilly, 2001; Matus-Amat et al., 2004; Stote and Fanselow, 2004; Rudy, 2009). Phenomenologically, fear becomes conditioned to a cue that warns of shock any time that the cue occurs. But fear of context does not develop if shocks occur too soon after an animal is introduced into a situation; the shock must be delayed by at least some 30 s, and full conditionability does not develop for several minutes (the so-called “immediate shock deficit”) (Blanchard et al., 1976; Fanselow, 1986, 1990; Landeira-Fernandez et al., 2006).

That there should be substantial differences between cue and context fear conditioning is not surprising, because learning to fear a context presents the nervous system with problems that do not occur during conditioning to simple warnings of a US. Contexts are defined by stable configurations or “conjunctions” of elementary cues (referred to here as “attributes”) that are present over extended periods of time. It can be expected that brains come to a learning situation pre-wired to represent the simple cues that, at least in the laboratory, are usually used as CSs for imminent USs. Such cues may be made to evoke fear simply by strengthening synapses between these representation neurons and amygdala fear-producing cells (see Blair et al., 2001).

However, it cannot be expected that neurons representing novel contexts, qua contexts, pre-exist. Neurons that innately represent the simple attributes which compose a context might of course come to evoke fear, but conditioning based on them would have unsatisfactory properties: Animals can presumably perceive only a small subset of a context's attributes at any one moment, and at each new entry to a given context, somewhat different attributes are likely to be sampled. Conditioning established to the small set of attributes that happened to be represented neurally at the moment of US occurrence would not produce fear during a different sampling of the same context. Therefore, stable contextual fear conditioning would seem to require the prior creation of a neural representation of the to-be-conditioned context that is constant despite variable samplings of the context's attributes. Also, there are likely to be many attributes of a context that are common to multiple contexts; if context fear were evoked by attributes *per se*, the existence of common attributes might well promote non-adaptive over-generalization. These problems can be avoided if conditioning occurs to a representation that is activated by a conjunction of contextual attributes rather than the individual attributes themselves. According to a variety of lines of current thinking, the role of the hippocampus in learning is to rapidly create conjunctive representations of combinations of simpler cues that then act as the neural stimulus for learnt contextual fear as well other kinds of learning (Fanselow, 1986, 2000; Kiernan and Cranney, 1992; Rudy and O'Reilly, 2001; Matus-Amat et al., 2004; Stote and Fanselow, 2004; Rudy, 2009).

Current theoretical thinking about the role of the hippocampus in learning stems from the work of Marr (1971). He proposed that, for a variety of reasons, the cortex learns slowly. But obviously animals must be able to learn some things rapidly. He argued that the intrinsic connectivity of the hippocampus and its connections to the cortex were consistent with the hypothesis that the hippocampus rapidly stores information about cortical patterns of activity. It does this, he suggested, in such a way that any sufficient fraction of the cortical activity pattern that was present at learning can evoke both the complete hippocampal pattern and, via that, the full cortical pattern that led to the learning. The fixed hippocampal patterns of Marr and followers theories (Marr, 1971; Skaggs and McNaughton, 1992; O'Reilly and McClelland, 1994; McClelland et al., 1995; O'Reilly and Rudy, 2001; Scharfman, 2007; Myers and Scharfman, 2011; Ketz et al., 2013) are just what are needed for representations to drive contextual fear responses. They are constant despite partial and variable sensory input, and since they are relatively non-overlapping (“orthogonal”), even for quite similar contexts, they would not lead to untoward generalization.

However, the fact that the nervous system does not receive information about all the attributes of a context at once means that over time there must be a continual evaluation and re-evaluation of the evidence as to whether the set of contextual cues so far sampled is from a familiar context or from a new context for which a representation should be established. This is essentially a Bayesian inference problem. The present model assumes a cortical-hippocampal circuit fairly similar to those of Marr and his followers. But it uses the Bayesian weight of evidence (Kass and Rafter, 1995; Gallistel, 2009) that the current context is or is not a known one to control learning within the hippocampal/cortical circuit and the hippocampus-amygdala pathway. We thus refer to the model as the Bayesian Context Fear Algorithm/Automaton (BACON).

## Methods

Most details not needed to understand Results are deferred to Supplementary Material.

### Scope

The goal of this work was to create a rational and neurally plausible algorithm for context fear conditioning. The development over time of hippocampus-independent engrams (systems type consolidation), was not considered but it is planned to address it at a later time.

### BACON neurons

The resting potential of BACON neurons is taken as zero, and membrane potentials (*V*) are specified relative to this baseline. Active synapses are thought of as causing post-synaptic conductances for specific ions. Excitatory input moves V toward an excitatory reversal potential *E*. Active synaptic inputs to a neuron cause post-synaptic conductances given by *G* = A ▪ W where *G* is conductance relative to membrane leakage conductance, *W* the synapse's strength or “weight,” which can vary from zero to any positive value, and *A* the activity level (firing rate, or “activation”) of the presynaptic neuron. The net depolarization of a neuron receiving both excitatory and inhibitory input is given by:

(1) $$V=\left(\frac{Ge\cdot E}{1+Ge+Gi}\right)$$

where Ge is total excitatory and Gi total inhibitory conductance.

Some BACON neurons are binary, firing at a maximum rate of unity when V is above some threshold value. For the remainder, activation rises linearly from zero starting at some threshold voltage (thrsh) and reaching a maximum of unity at a higher voltage (mxat). We refer to this as a “linear sigmoid activation function,” which we write as follows:

(2) Activation=linsig(V|thrsh,   mxat).

**Figure 1:**
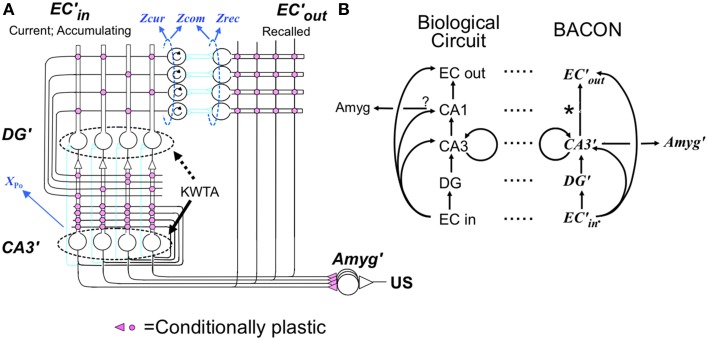
**Cortex-hippocampus-amygdala circuit (A) basic circuit**. Explained in text. Pink synapses subject to LTP in appropriate modes. Circular arrows in EC'_in_ cells indicate working memory properties. **(B)** Comparison between biological and BACON circuits. *indicates absence of a CA1 analog in BACON, as various simplifications made its presumed functions redundant (see Results).

The basic cortical-hippocampal-amygdala circuit of BACON is shown in Figure 1, which will be explained in Results. Table 1 gives salient properties of the various classes of neurons. As explained in Results, algorithm neuron types are named for their intended biological parallels but with a prime mark added to distinguish them from their biological counterparts.

**Table 1:** **Properties of BACON neurons**.

**Cell type**	**Properties**
EC'_in_	Binary. Working memory properties-see Results
EC'_out_	Binary. Thrsh = *ThrshCtx*
DG'	KWTA activation rule in creation and update modes; rate coded in recall mode
CA3′	KWTA activation rule
Amyg'	Rate coded

Most of the computations done by the algorithm are explained in logical or mathematical terms. Actual neural circuitry that could underlie them is unknown, but circuitry that could compute the Bayesian Weight of Evidence, which is central to the model, and circuitry that could use it to control hippocampal and amygdala function are sketched out to demonstrate that they could be carried out by neurons. Ways of carrying out arithmetical operations such as subtraction, multiplication, exponentiation, etc. assumed done within this hypothetical circuitry and ways of implementing K-Winners-Take-All control of neural firing, used extensively in BACON, are discussed in the Supplementary Material (Topic H).

The values and definitions of BACON's parameters are summarized for reference in Table 2.

**Table 2:** **BACON parameter definitions and values**.

Numbers of EC'_in_ and EC'_out_ cells	*N_Ctx_*	1000
Numbers of DG and CA3′ cells	*N_Hipp_*	10,000
Number of attributes per context	*N_A_*	100
Number of attributes common to all contexts (“General” attributes)	*N_Gen_*	50
Number of EC'_in_ neurons innervating each DG neuron	*F*	60
Number of winners of hippocampal KWTA calculations	*K*	60
Minimum number of cells that must be active in CA3 pattern _CA3_Ptrn_o_ for recurrent collateral input to be computed	*K_0_*	15
Minumum number of EC'_in_ cells that must be active for a hippocampal representation to be created	*Z_0_ (minZ0)*	45
Numer of effective inputs needed for a EC'_out_ cell to fire	*ThrshCtx*	42
Minimum Bayesian weight of evidence for conditioning to occur	*B_old_*	3
Negative Bayesian weight of evidence sufficient for representation creation	*B_new_*	−3
Minimum Bayesian weight of evidence for addition of attributes to an existing hippocampal representation	*B_add_*	15
Bayesian weight of evidence making representation “probably valid”	*B_pv_*	3
Amygdala learning rate parameter	α	0.025
Factor multiplying excitation produced in CA3′ via direct pathway	*dpf*	Varied

### Computations

A flow diagram of the computations done by BACON is provided in Supplementary Material (Topic G).

## Results

The goal of this work was to construct a biologically plausible algorithm/automaton for context fear conditioning. It is assumed that upon entering a context its attributes are observed (“sampled”) serially in random order without replacement over an extended period of time and are held in a working memory. As attributes are sampled, we wanted the automaton to compare them to the recalled attributes of previously experienced contexts and use the comparison to decide whether the context is a known one, in which case existing associations to it should be expressed and new ones made, or a novel one, in which case a neural representation of it should be created and further associations made to that. The algorithm we have created deals with these matters using analogs of cortical, hippocampal, and amygdala structures; we name these for their biological counterparts *appended with a prime (') mark*.

### The basic cortical-hippocampal-amygdala circuit

#### Structure

Figure 1A presents BACON's basic circuit, which as indicated in Figure 1B, is formally similar to its biological counterpart. As in most models inspired by Marr's original work, BACON's hippocampal circuitry is designed to construct a cortical “image” of previously experienced events when probed by a sufficient fraction of the image. It does this by creating a sparse, random hippocampal representation of the event that can be fully activated by fragmentary cortical images, and that can then in turn reactivate all those cortical neurons that were active at the time that the hippocampal representation was created. In designing BACON's hippocampus, we attempted to implement the presumed basic operational principles of the biological hippocampus in as clear and simple a form as possible. We did not include features in the name of fidelity if they did not seem useful to the task at hand.

#### Entorhinal cortex

Cortical input is presented to BACON's hippocampus' by an entorhinal cortex (EC') that consists of *N_Ctx_* (=1000) input (EC'_in_) neurons, each of which innately represents one of the possible contextual attributes that the automaton can detect. Hippocampal output back to cortex is via a corresponding set of output (EC'_out_) cells. Each context is composed of *N_A_* (=100) attributes of which a number, *N_Gen_* (=50) are general to all contexts. When an attribute is sampled the EC'_in_ cell that represents it becomes active and remains so for the duration the visit to that context. Neurons that remain active during a working memory task have in fact been found in entorhinal cortex (e.g., Suzuki et al., 1997; Young et al., 1997; Schon et al., 2015).

#### Dentate (DG')

DG' consists of a large number, *N_Hipp_* (=10,000), of projecting neurons that during representation creation recode the EC'_in_ pattern of activity into a sparse, relatively non-overlapping form (as in O'Reilly and McClelland, 1994). Each DG' cell is innervated, via Hebbian synapses, by *F* (=60) EC'_in_ neurons. *All of BACON's Hebbian synapses are totally ineffective until potentiated*. During representation creation, firing of the projecting DG' neurons is subject to regulation by an inhibitory network that allows only a specified number of the most excited cells to fire (“K-Winners Take All” (KWTA) behavior). When operative, this allows *K* =60 of DG's 10,000 cells to fire. At other times, DG' cells fire at a rate proportional to their excitation.

#### CA3′

As in the biological hippocampus, CA3′ cells receive input from EC'_in_ both via DG' (the “indirect” pathway) and via a direct pathway from EC'_in_. As was the case for DG', each CA3′ cell is innervated by *F* randomly chosen EC'_in_ neurons, but these are chosen independently of the DG' innervation. In the biological hippocampus each CA3 cell is innervated by a small number of dentate cells, and the synapses, which appear from morphology to be strong, show some non-Hebbian sort of plasticity of uncertain function (Bortolotto et al., 2003; Reid et al., 2004). In BACON this arrangement has been simplified (as considered in Discussion); each CA3′ cell is innervated by a single DG' neuron via a non-plastic synapse whose strength can be modulated. CA3′ cells, like DG' cells, are subject to KWTA regulation of their firing; the number of cells allowed to fire is the same as in DG'. In the biological hippocampus, each CA3 cell recurrently innervates other CA3 cells via Hebb synapses. This is thought to form an auto-associative memory that binds together the member-neurons of each representation so that when a sufficient subset is activated the rest will follow (e.g., Gardner-Medwin, 1976; McNaughton and Morris, 1987; Treves and Rolls, 1994). In the biological hippocampus each CA3 cell innervates only a portion of others; in BACON we have for simplicity allowed full innervation.

#### CA3′, CA1′, and EC'_out_

Entorhinal cortex receives from hippocampus representations of contexts that consist of the activity of essentially random sets of cells. It is thought that these are able to recreate entorhinal output replicas of the entorhinal input patterns that they encode because during representation creation potentiation develops between cells of the ‘random’ hippocampal representation and EC output cells corresponding to EC input cells that are active (e.g., Treves and Rolls, 1994).

It seems as if this function would be best served if each CA3 cell were to directly innervate each entorhinal output neuron via a Hebb synapse, which is exactly how BACON is constructed. However, fan-ins and fan-outs of most biological neurons are too low for each real CA3 neuron to completely innervate every EC output cell. The interposition of CA1 between CA3 and EC in the biological Hippocampus is thought to mitigate this limitation by recoding the CA3 output into a less sparse form in CA1 so that every EC output cell can receive some innervation that is specific to each encoded pattern (e.g., Kesner and Rolls, 2015). A second function of biological CA1 is thought to be to help complete representations that, because of incomplete innervation in the recurrent collateral network, were incompletely reconstructed by CA3 auto-associative mechanisms (Treves, 1995). However, because in BACON we allow complete innervation within the CA3 recurrent collateral system, this function is also unneeded. Since a CA1′ would therefore have served no useful function in BACON, it was for simplicity omitted.

#### Amygdala'

Although we assume that hippocampal circuitry evolved to allow reconstruction of the neocortical activity that occurred during past events, *it is to the hippocampal code proper and not to the cortical reconstruction of a context's attributes* that fear becomes conditioned in BACON's amygdala'. We constructed BACON in this way for three reasons: (i) We believe that it is consistent with the weight of available evidence (as discussed in Krasne et al., 2011). In particular, it appears from the immediate shock deficit that fear does not readily become conditioned to the attributes of a context *per se*. (ii) Hippocampal representations of very similar contexts overlap much less than do cortical ones, which makes discriminations easier to learn. (iii) Hippocampal representations are (by construction) constant whereas cortical ones change as more is learned about a context; changing representations would lead to unwanted variations in expression of previously conditioned fear.

The actual (biological) pathway via which information about active hippocampal representations reaches the amygdala from the dorsal hippocampus is a matter of current research. Based on various lines of anatomical, and more recently labeling and optogenetic data, ventral hippocampus as well as various cortical structures have been suggested as possibly being on the relevant pathway Maren and Holt, 2004; Fanselow and Dong, 2010, Cowansage et al., 2014; Tanaka et al., 2014; Jin and Maren, 2015. Whatever the final resolution of this matter, BACON assumes (as said above), that it is the dorsal hippocampal representation (*or downstream, possibly cortical, versions of it*), and not cortical attribute-representing neurons themselves, to which contextual fear responses become conditioned.

CA3′ innervates amygdala' fear-producing cells via Hebb synapses, and conditioned fear develops when synapses between active CA3′ cells and amygdala' neurons that are depolarized by an unconditioned stimulus become potentiated.

#### Operational modes

The above features of BACON are all equivalent to, or slight modifications of, conventional ideas about the role of the hippocampus in creating and utilizing representations of complex stimuli and in context fear conditioning. However, BACON departs from most conventional thinking in that it includes circuitry conceptualized as outside the hippocampus that makes evidence-based decisions as to whether the automaton's current context is or is not a familiar one, and depending on its conclusions, configures the hippocampus and amygdala appropriately for representation *creation* or *recall* as well as controlling *conditionability* and the *expression* of previously conditioned fear. This control is exerted by enabling or disabling the possibility of Hebbian potentiation, modulating the strength of certain synapses, and turning on or off the KWTA capability of those regions that are potentially subject to it. The idea that hippocampal circuitry is subject to task-based reconfiguration has precedents in the work of O'Reilly, McClelland, Rudy, and Hasselmo (e.g., O'Reilly and McClelland, 1994; Hasselmo et al., 1995; O'Reilly and Rudy, 2001; Hasselmo and Sarter, 2010).

#### Representation creation

**Figure 2:**
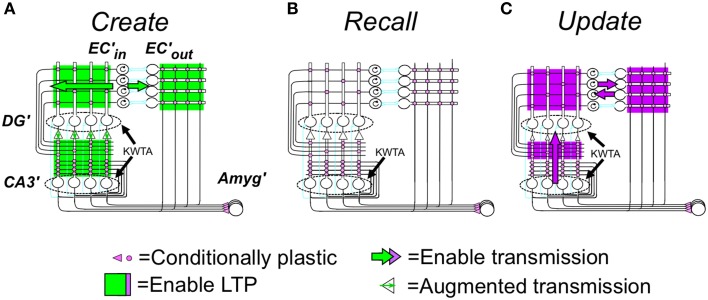
**(A)** Creation, **(B)** Recall, and **(C)** Update modes. Explained in text but some details follow: **(A)** Creation mode details: (1) Noise in the system prevents any ties of excitation of DG' cells during representation creation, so *exactly K* DG' cells fire. (2) Potentiation of synaptic weights of EC'_in_ synapses on each DG' or CA3′ cell occurs in such a way that the weights of potentiating synapses sums to one; such normalization of weights is needed so that the representations of contexts created with larger numbers of EC'_in_ neurons active do not dominate in later KWTA competitions. Other synapses potentiate in one step to fixed weights. **(B)** Recall mode details: (1) During production of the pre-recurrent CA3′ activity pattern _CA3_Ptrn_o_, noise in the system prevents any ties of excitation, so *at most*, *exactly K* DG' cells fire. If the number of active neurons in _CA3_Ptrn_o_ is less than *K_0_*, CA3′ activity is extinguished until the next attribute is sampled. (2) The excitation evoked by recurrent collateral activity causes the *K* most excited CA3′ cells to fire to produce _CA3_Ptrn_1_ and higher order patterns with ties *not* being broken, so more than *K* cells may fire; allowance of more than *K* cells is necessary for convergence to the correct pattern (see Supplementary Material, Topic B). (3) When evidence for two patterns is exactly equal, _CA3_Ptrn_Fnl_ will contain more than K active cells, and further downstream actions will be suppressed. **(C)** Update mode specifics: (1) EC'_in_–EC'_out_ transmission is enabled bi-directionally (purple top block arrows) so that the representations of all currently sampled and recalled attributes will be active in both EC'_in_ and EC_out_. (2) Transmission in a powerful “backward” pathway from CA3′ to DG' (up-pointing purple block arrow) and KTWA control of DG' firing are enabled. As a result, DG' activity mirrors _CA3_P_fnl_, which will be the complete representation with no other cells active. (3) EC'_in_, DG', CA3′ and CA3′-EC'_out_ synapses become LTP-susceptible, and all coactive EC'_in_-DG' and CA3′ synapses and all CA3′-EC_out_ synapses become potentiated if they are not already so. As in representation creation, synaptic weights of active EC'_in_ synapses on each active DG' and CA3′ cell are normalized to a sum of one. *Information flow in the biological dentate-CA3 pathway is usually considered to be only in the forward direction, and therefore the above use of “backward” transmission may seem biologically implausible. However, a backward pathway from CA3 to dentate has in fact been described (Scharfman, 2007; Myers and Scharfman, 2011), and there is also some evidence for retrograde chemical signaling across synapses in the forward pathway. Contractor et al. (2002) that could imaginably trigger LTP at presynaptically active synapses on dentate neurons innervating active CA3 cells. We therefore do not consider this computationally motivated feature biologically implausible.

When BACON decides it is in a novel context, it configures the circuitry of Figure 1 for representation *creation* (Figure 2A). This includes (1) enabling the plasticity of all the Hebbian synapses of hippocampus and cortex, (2) enabling transmission at all EC'_in_-DG', EC'in-CA3′, and EC'_in_-EC'_out_ synapses (irrespective of the state of potentiation of the plastic synapses), and (3) enabling the KWTA circuitry of DG' (the KWTA circuitry of CA3′ is always operative). Finally, the strength of transmission at DG' to CA3′ synapses is up-modulated sufficiently so that the *K* cells that will fire in CA3′ will be the partners of the *K* winners of the DG' KWTA competition and not be affected by direct EC'_in_ input.

Under these conditions the *K* DG' cells that are most strongly excited by the active EC'_in_ attribute cells fire and drive their CA3′ partners. The synapses between all active EC'_in_ cells and the active DG'-CA3′ partners then become potentiated. It should be noted that this method of determining which DG' and CA3′ cells come to represent a context ensures that the representations will be composed of those DG'-CA3′ “dyads” whose DG' member is most strongly *innervated* by the EC'_in_ cells representing the attributes that had been sampled up to the moment of the representations creation.

Once the *K* most excited CA3′ neurons fire, the activity of each active CA3′ cell propagates into its dendrites and collaterals, and the recurrent synapses between one active CA3′ cell and another potentiate. The *permanent* representation of the current context has at that point been established as the active set of DG'-CA3′ dyads. It is the potentiated recurrent collateral synapses that bind together the set of cells that comprise a representation, and it is the potentiated synapses in the direct and indirect pathways that are responsible for the ability of a given context's attributes to call out its proper representation. Finally, the synapses between the active CA3′ representation neurons and the set of active EC'_out_ attribute cells that are being driven by the active EC'_in_ cells become potentiated so that in the future when this representation becomes active it will cause those same EC'_out_ cells to fire.

It should be noted that, as will be explained below, representation creation generally occurs when only a portion of a context's attributes have been sampled. Therefore, even if two contexts are extremely similar, their representations will be created with somewhat different sets of EC_in_ attribute cells active.

In some biological experiments to be discussed, DG' was inactivated during encoding. If this is done in BACON, it will be the KWTA properties of CA3′ operating on input via the direct path that will determine which CA3′ neurons comprise the new representation.

#### Recall

In the resting state, the cortical and hippocampal parts of Figure 1 are configured for *recall*. In this mode (Figure 2B) no synaptic plasticity is enabled, and plastic synapses operate according to their current state of potentiation. When in recall mode, the ratio of the strength of direct to indirect pathway synapses on CA3′ cells is a parameter *dpf* (direct path factor), the effect of whose value we study below. KWTA control is disabled in DG' during recall, with the result that cells there fire at rates proportional to their excitation; however KWTA control is always operative in CA3′.

Under these conditions active EC'_in_ neurons excite DG' and CA3′ cells via any previously potentiated synapses. The DG' cells fire in proportion to their excitation, exciting CA3′ cells proportionately, and the direct input from EC'_in_ sums with the excitation from DG'. The KWTA rule then determines CA3′ firing. The pattern of activity at this stage we call _CA3_Ptrn_o_.

Once sampling of a context's attributes is fairly far advanced, the strongly excited cells included in _CA3_Ptrn_o_ will mostly be ones that were active when a context's representation was created. However, earlier in sampling, when only a modest fraction of the total set of a context's attributes have been sampled, it can easily occur that representation cells that do *not* represent the current context will be part of _CA3_Ptrn_o_. This happens because, as mentioned above, when two contexts have attributes in common (which they always do, since *N_Gen_* is 50% of *N_A_*), which of these get encoded when their representations are created is usually somewhat arbitrary. Therefore, during recall in one of the contexts, shared attributes that were encoded for the other context may well be the ones first sampled and may cause representation cells unique to this (incorrect) context to be activated.

Next, _CA3_Ptrn_o_ will generate a new pattern of CA3′ excitation via the potentiated synapses of the recurrent collateral system, and the *K* most excited cells will again fire. This process repeats a specified number of times until a final pattern referred to as _CA3_Ptrn_Fnl_ results. In BACON, where each CA3′ cell recurrently innervates each other one, a _CA3_Ptrn_o_ in which a plurality of the cells are part of some representation will, after two iterations generate a _CA3_Ptrn_Fnl_ in which the cells active are the *K* representation cells for that context and no others (so long as not too many extremely similar contexts have been encoded – see Supplementary Material, Topic B). If no context's representation cells are in a plurality, _CA3_Ptrn_Fnl_ will consist of more than *K* cells (a summation of more than one representation), and in that case no downstream activity will be produced (see Figure 2, caption and Supplementary Material, Topic B).

Finally, _CA3_Ptrn_Fnl_ excites EC'_out_ cells via any potentiated synapses, and those EC'out neurons that get excited by a sufficient proportion (*ThrshCtx*) of the active CA3′ cells fire.

#### Representation updating

If, after sampling a new attribute, BACON determines that it has correctly identified the context it is in, or it has created a new representation during the current session, it adds the newly sampled attribute to the currently active contextual representation. It does this by going into *update* mode (Figure 2C). In this mode, once _CA3_Ptrn_Fnl_ has been activated, backward transmission from CA3′ to DG' is enabled so that DG' mirrors CA3′ activity. Transmission between EC'_in_ and EC'_out_ is also bi-directionally enabled so that the EC'_in_-EC'_out_ pairs corresponding to all currently sampled and recalled attributes are active. And plasticity is enabled at EC'_in_-DG' and -CA3′ and CA3′-EC'_out_ synapses. These actions associate the newly sampled attribute with the current representation, while keeping constant the set of DG'-CA3′ pairs that constitute the representation.

#### Context fear conditioning

Contextual fear conditioning in BACON is due to potentiation of CA3′-amygdala' synapses and is explained at the end of the next section.

### Bayesian control of operation mode of hippocampo-cortico-amygdala circuitry

Based on comparisons of current EC'_in_ activity, the status of which depends on the outcome of BACON's sampling of the current context so far, and EC'_out_ activity, which depends on the automaton's cumulative past experience, BACON determines the Bayesian weight of evidence (Kass and Rafter, 1995; Gallistel, 2009, here denoted as *B_Rep_*) that an active hippocampal representation really is that of the current context. It then uses this value to decide whether to create a new representation or update the current one, whether to permit fear conditioning if a US occurs, and whether to allow expression of conditioned fear.

#### Evaluating representation validity

The calculation of *B_Rep_* is based on (1) the number of attributes of the current context that have been sampled and have active representations in EC'_in_ (referred to as *Z_cur_* –for “*Z_current_*”), (2) the number of attribute representations activated in EC'_out_ by the current hippocampal representation (*Z_rec_*–for “*Z_recalled_*”), and (3) the number of active attribute representations in common between EC'_in_ and EC'_out_ (*Z_com_*–for “*Z_common_*”). *B_Rep_* is defined as:

(3) $$B_{\text{Rep}}\left(Z_{com}|Z_{cur},Z_{rec}\right)=\log_{10}\frac{\text{P}\left[Z_{com}|\text{Same},Z_{cur},Z_{rec}\right]}{\text{P}\left[Z_{com}|\text{Diff},Z_{cur},Z_{rec}\right]}$$

where “Same” means that the active contextual representation is that of the actual context and “Diff” means that it is not.

**Figure 3:**
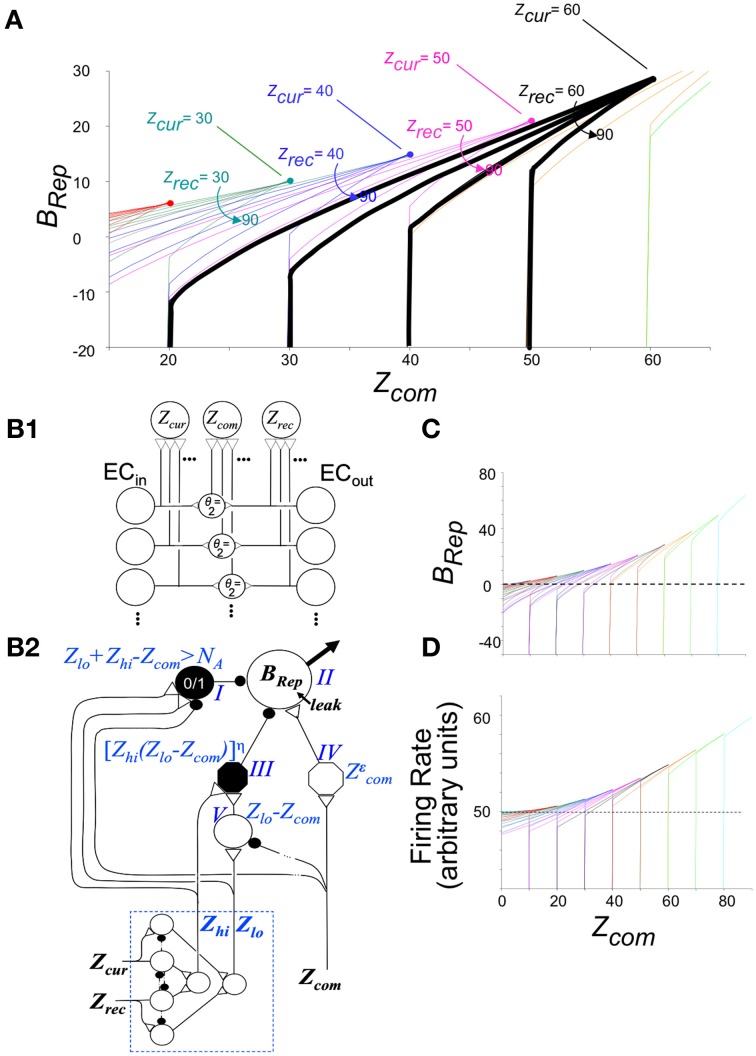
**Bayesian Weight of Evidence, *B_Rep_*: Exact calculations and neural circuit that emulates them**. **(A)** Exact values of *B_Rep_* are plotted as a function of *Z_com_* for a variety of values of *Z_cur_* and *Z_rec_*. The values for a given value of *Z_cur_* are all in the same color. The values for *Z*_rec_ = 60 and *Z_cur_* = 60–90 are made bold black to highlight one set of values for reference when reading the relevant text. **(B)** Neural circuit that approximates exact calculations. **(B1)** Circuit for getting rate-coded inputs *Z_cur_, Z_rec_, and Z_com_* to the rest of the circuit. It is assumed that *Z_cur_*, *Z_rec_*, and *Z_com_* fire at rates proportional to their number of active inputs; interneurons are binary cells that fire when both their inputs are active (i.e., threshold, θ = 2). **(B2)** *B_Rep_* computing circuit. Because *B_Rep_* is symmetrical with respect to *Z_cur_* and *Z_rec_* (i.e., their values can be exchanged without effect), these are transformed by the module at the lower left to *Z_lo_* and *Z_hi_* (the lower and higher of the two). Neurons of the computational module proper are labeled I–V for reference. Those represented as octagons do arithmetic that may itself require a small circuit (see Supplementary Material). Neuron II, which codes *B_Rep_* as a firing rate, is the output neuron of the circuit. It is given a leakage conductance that elevates its baseline potential and firing rate above zero so that that these can both increase, to convey confidence in the validity of the currently active representation, and decrease, to convey confidence that it is invalid. The remainder of the circuit was constructed to approximate the exactly calculated value of *B_Rep_* as a function of *Z_com_, Z_lo_*, and *Z_hi_* shown in **(C)**. When *Z_com_, Z_rec_*, and *Z_hi_* of the exact curves are equal, *B_Rep_* increases from zero as an accelerating function of the value of *Z_com_*, forming a curve that is the upper envelope of the set of full *B_Rep_* curves. This upper envelope is emulated by the output of neuron IV, which transforms Zcom into a power function of *Z_com_* with the exponent being chosen so that the firing rate of II will approximate the upper envelope of the exactly calculated curves. By definition, *Z_com_* cannot be greater than *Z_lo_*. For a given value of Z_lo_, *B_Rep_* is on the above-described envelope when *Z_com_ = Z_lo_ = Z_hi_*. It decreases as *Z_com_* decreases, at a rate that is greater, the greater *Z_hi_*, as seen in **(C)**. This behavior is emulated if neuron II is subject to divisive inhibition that is proportional to a power function of *Z_hi_*(*Z_lo_* − *Z_com_*). Neuron III provides this inhibitory input; it receives excitatory input from both *Z_hi_* and neuron V, whose output is proportional to *Z_lo_* − *Z_com_*. The output of neuron III is proportional to a power function of the product of its inputs. If an active hippocampal representation is valid, then *Z*_lo_ + Z_rec_ - Z_com_ must be less than or equal to *N_A_*. If this condition is violated, the current context cannot be that currently represented by the hippocampus, and *B_Rep_* will be infinitely negative, as seen in the exact calculations of **(A,C)**; in the circuit of **(B)** neuron I fires when *Z*_lo_ + Z_hi_ = Z_com_ > N_A_ and massively inhibits neuron II. **(C)** Exact values of *B_Rep_* calculated from combinatorial formulas. Color coding as in **(A)**. **(D)** Firing rate of neuron II. Note that it emulates **(C)** quite well.

Figure 3A graphs *B_Rep_* as a function of *Z_com_* for a range of values of *Z_cur_* and *Z_rec_*; the combinatorial formulas used to calculate these values are given in Supplementary Material (Topic C). High positive values mean that the specified degree of communality between EC'_in_ and EC'_out_ activity makes it very likely that the actual current context is in fact the one that was present when the currently active hippocampal representation was created. A large negative value indicates that the actual current context is not the one currently represented by the hippocampus. A value of zero means that there is no basis for believing either hypothesis. We note that *B*_Rep_ = 0 if EC'_out_ remains silent, as it does when the number of cells active in the initial CA3′ pattern of the computation cycle (*X_Po_*) is less than *K_0_* (the number active cells in _CA3_Ptrn_o_ needed to activate the recurrent collaterals).

The relationships shown in Figure 3A conform well with logical or intuitive judgments based on the same information. Consider for example the bold black curves for *Z*_cur_ = 60. (1) For any given number of current and recalled attributes, the greater *Z_com_*, the more likely it is that the currently active representation is in fact that of the current context, and correspondingly, *B_Rep_* increases as a function of *Z_com_*. (2) The more one recalls about the context one thinks one is in, the more strongly a low value of *Z_com_* implies that one is actually somewhere else, and conversely. Consistent with this, for a given value of *Z_com_*, *B_Rep_* decreases as *Z_rec_* increases. (3) If the number of currently observed attributes that are different from ones remembered (*Z_cur_–Z_com_*) plus the number remembered (*Z_rec_*) is greater than the number of attributes there are per context (*N_A_*), then the current context cannot be valid. Consistent with this, *B_Rep_* goes precipitously negative if *Z_cur_ - Z_com_ + Z_rec_ > N_A_*.

**Figure 4:**
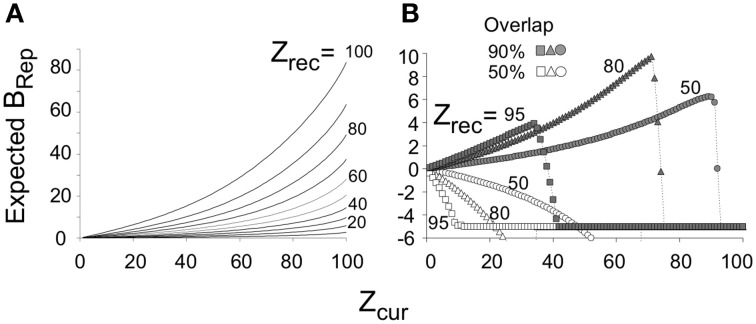
**Expected *B_Rep_* values as a function of *Z_cur_* for various values of *Z_rec_***. **(A)** *Expected B_Rep_* as a function of *Z_cur_* when BACON is tested in a context that is more or less well-known (differing *Z_rec_* values). **(B)** If BACON is placed in a novel context that is somewhat similar to one for which there is a representation, the representation of the known context will become active. Expected *B_Rep_* for this representation as a function of *Z_cur_* is shown for several degrees of similarity between the two contexts and several levels of *Z_rec_* for the encoded context. When the current context and the familiar one are not very similar, *B_Rep_* goes negative from the very start of the session and does so more rapidly, the better known (i.e., the greater the *Z_rec_* of) the familiar context. When the two contexts are similar, *B_Rep_* also eventually goes negative and does so sooner the more is known about the familiar context. However, especially at intermediate values of *Z_rec_, B_Rep_* for the previously familiar context first becomes quite large.

We will later discuss experiments in which animals are pre-familiarized to contexts in which they will subsequently be conditioned. Intuitively, it would be expected that the more attributes of the context were previously learned, the sooner *on average* the current context could be identified with assurance. Figure 4A plots *B_Rep_* values for the theoretically predicted average (referred to as “expected”) *Z_com_* for a test in the conditioning context; we refer to *B_Rep_* so calculated as *Expected B_Rep_*. The greater *Z_rec_* due to experience with the context in prior sessions, the faster *Expected B_Rep_* rises as more attributes are sampled in the test session.

#### Control of representation creation and updating

This section describes the logical rules used for controlling the state of the basic circuit; neural circuitry for implementing these rules is discussed separately.

We define certain levels of *B_Rep_* (*B_old_*, *B_new_*, *B_add_*, and *B_pv_*) that determine the mode of operation (Figure 2) of the basic circuit, sometimes along with other conditions. *B_old_* is taken as the lowest level of *B_Rep_* that provides reasonable but not foolproof evidence that the currently active hippocampal representation is in fact the one that was created for the current context (i.e., is “valid”). As explained in the next section, this is the level at which conditioning begins to become possible. If *B_Rep_* exceeds this value, we speak of the current context as probably being “known” or “familiar.” *B_add_* is taken as the level of *B_Rep_* at which the automaton is virtually certain that the current representation is valid, and at which attributes are therefore allowed to be added to active representations (i.e., updating mode is entered). We note that it is crucial that a representation not be updated unless it is almost certain that it is valid, because adding invalid attributes to an existing representation would render it valid nowhere, and any conditioning that had occurred to it would be lost to effective usage. Therefore, *B_add_* is made much higher than *B_old_* (Table 2). *B_new_* is the negative value of *B_Rep_* below which it is deemed highly unlikely that the current representation is valid (i.e., BACON is probably in an unknown context for which it has no representation).

When *B_Rep_* falls below *B_new_*, creation mode (Figure 2A) may be entered and a new representation established, but two additional conditions must be satisfied:

1. New representations should be created only when enough of the current context's attributes have been sampled so that, once learned, they will suffice to allow recognition of the context on future occasions; we call this number *Z_0_*. The minimum useful value for *Z_0_* would be that for which *B_Rep_* reached *B_old_* once all *N_A_* attributes of the context were sampled. With the parameters used here this would be *Z_0_*= 10. However, if *Z_0_* were so low, recognition of the context on future occasions would require that every attribute of a context have been sampled before reasonably confident recognition could occur, which would presumably take a maladaptively long time to be completed. In fact, we will argue below that for our parameters, *Z_0_* should be at least 45, which is the value that we adopt for the simulations of this paper.
2. If placed in one of two quite similar familiar contexts, vicissitudes of attribute sampling early in the session are likely to cause the representation of the incorrect context to be activated intermittently until enough information about the actual context has been acquired so that the correct representation becomes continuously active. Such invalid representations can sometimes cause *B_Rep_* values sufficiently negative to trigger creation of a new representation, which would then be a second (and unwanted) representation for the same context. To reduce the likelihood of this happening, representation creation is suppressed if during the session, some representation has generated a *B_Rep_* sufficiently positive that it is likely to be the actual representation of the context; the degree of positivity required is *B_pv_* (“pv” for “probably valid”), and for the simulations of this paper this was set equal to *B_old_*. This rule helps prevent unwanted representation creation; however, if the automaton is placed in a *new* context that is quite similar to a familiar one, the representation of the familiar context is likely to be activated and may generate a quite high *B_Rep_* value (see Figure 4B and associated discussion). When this happens, the above rule, if unmodified, would prevent a representation of this new context from being created. However, if the context is novel, currently observed attributes and recalled attributes associated with existing representations will eventually disagree sufficiently to generate *B_Rep_* values below *B_new_*, and once this has occurred for all representations that formerly dictated suppression of creation, the suppression is lifted and creation of the new representation is allowed.

Given the above rules, creation of a new representation will usually occur when an active representation generates a *B_Rep_* value below *B_new_*. However, if there is no existing representation for some context at least somewhat similar to the current one, the number of CA3′ cells initially activated (denoted *X_Po_*) may remain below *K_0_*, EC'_out_ remain silent, and *B_Rep_* remain zero, even after sampling considerable numbers of attributes. In such cases a new representation is created if the number of cells active in the pre-recurrent input activity pattern of CA3′ (*X_Po_*) is still less than *K_0_* by the time *Z_cur_* reaches *Z_0_*.

Finally, if a new representation has just been created, it is by definition valid for the remainder of the session; therefore updating occurs at every subsequent sample of the session.

#### Control of context fear conditioning and expression

#### Conditionability

Context fear conditioning in BACON is due to LTP of CA3′-Amygdala' synapses, which occurs at synapses between active CA3′ neurons and amygdala' cells when the amygdala' cells are depolarized by a US (Maren and Fanselow, 1995; Johansen et al., 2014). Obviously this cannot occur until a representation of the context has been created, but one would also not want it to occur unless there was reasonable evidence that the representation is valid.

**Figure 5:**
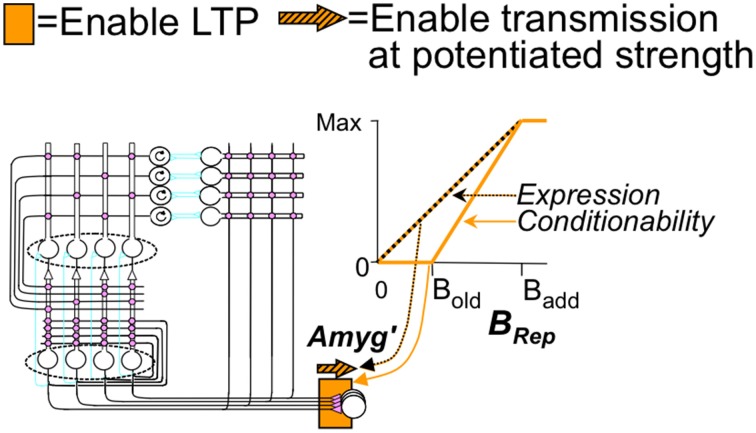
**Fear conditioning and expression modes**. Susceptibility to LTP (conditionability) and transmission at levels determined by established LTP are neuro-modulator controlled as shown. Explained in text.

**Figure 6:**
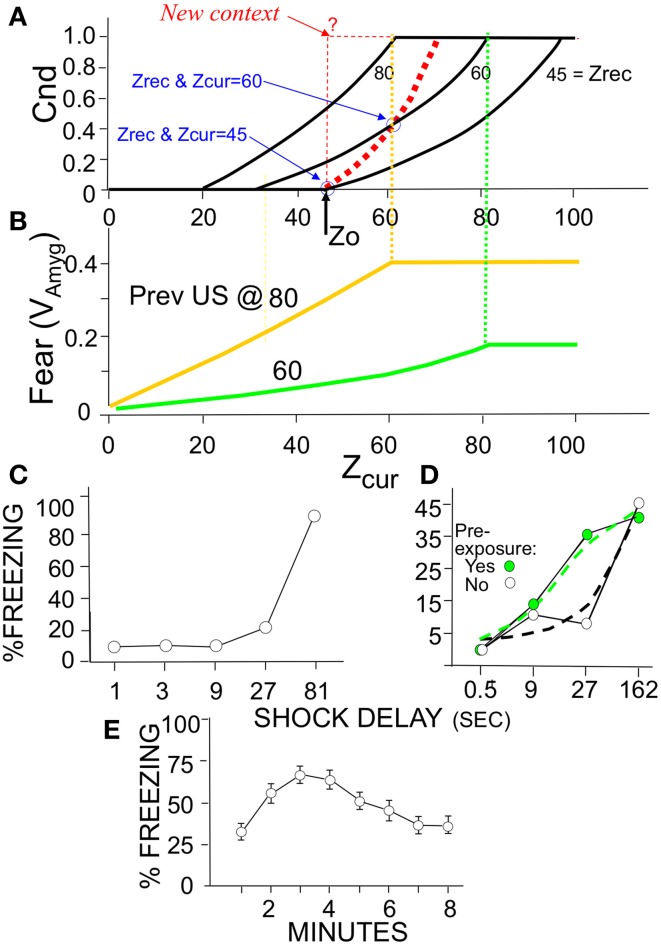
**Expected *Cnd* and Fear (*V_Amyg_*) as a function of *Z_cur_* and *Z_rec_* with corresponding animal data**. **(A)** Conditionability, as explained in Results. The black curves are for sessions entered with the specified *Z_rec_* values. For all curves *Expected Cnd* = linsig [*B_Rep_*(*Expected Z_com_|Z_cur_, Z_rec_*)*|B_old_, B_add_*]. The bold dashed red curve is for a session in which representation creation occurs at *Z_0_*; it is calculated in the same way as the other curves but taking into account that *Z_rec_* increases with each successive sample subsequent to representation creation; thus *Z*_rec_ = Z_cur_ (as indicated for the two circled values). The thin dashed red line corresponds to the empirically untenable possibility, discussed in the text, that *Cnd* rises to its maximum value as soon as a representation is created. **(B)** Fear as a function of *Z_cur_* for automata previously conditioned in a new context with a US after 60 or 80-samples and the session then terminated. Vertical dashed lines indicate that *Cnd* and Fear expression plateau at the same number of attributes sampled. **(C–E)** Empirical demonstrations of effects produced by BACON **(C)** Immediate shock deficit (Data from Fanselow, 1990, Figure 2). **(D)** Shortening of deficit due to pre-exposure (Redrawn data from Fanselow, 1990, Figure 3; dashed lines indicate an interpretation consistent with simulation data). **(E)** Gradual onset of freezing at start of session (Data from Wiltgen et al., 2006). Decline is due to extinction, which automaton does not emulate.

We thus make conditionability increase linearly as a function of the degree to which *B_Rep_* exceeds its value at *B_old_*, reaching a maximum of unity when *B_Rep_ = B_add_* (Figure 5—recall that *B_add_* corresponds to virtual certainty):

(4) Cnd=linsig  (BRep|Bold,Badd),

(see Methods for definition of linsig). We then calculate the increment in strength due to a US as:

(5) ΔStrength=α ▪ Cnd

where α is the amygdala learning rate parameter (Table 2). The black curves in Figure 6A show the growth of conditionability as sampling of contextual attributes progress throughout a session in a context for which a representation was previously created. The curves start rising from *Cnd* = 0 when expected *B*_Rep_ = B_old_. *B_Rep_* increases both earlier and faster with increasing *Z_rec_* because the more that is known about the context, the faster *B_Rep_* increases as information about the current context is acquired.

This much is straight-forward and we believe plausible. However, special issues arise when USs occur *during* sessions where representations were created.

One of these concerns the value of *Z_0_* (the minimum *Z_cur_* at which a representation can be created). We argued above that given BACON's parameters, *Z_0_* should be at least 10. However, if *Z_0_* were in fact 10 and we introduced BACON into a new and distinctive context, a representation for the context would then be created at sample 10. Since, as pointed out above, a representation is by definition absolutely valid for the remainder of a session in which it is created, conditionability should at least start to rise from zero at that point. However, suppose we were to remove BACON from the context right after the representation was created and then later re-introduce it. Since *Z_rec_* would now be only 10, all *N_A_* (=100) attributes would have to be sampled before *B_Rep_* rose to *B_old_* and conditioning became possible. That would leave us with the peculiarity that conditioning becomes possible after 10 samples in a fully novel context but only after 100 in one with which the automaton is already familiar. In order to prevent such an anomaly, *Z_0_* would have to be such that *Expected B_Rep_* on a session following an initial one that terminated just after *Z_0_* samples, would reach *B_add_* once this same number of samples were made in the latter session (i.e., *Expected B*_Rep_ = B_add_ when *Z*_cur_ = Z_rec_ = Z_0_). Reference to Figure 6A shows this to occur when *Z*_0_ = 45, which value we have used for the simulations of this paper.

The other issue concerns the *rate* at which *Cnd* should increase if the automaton is left in the context after creation of the representation. Basing *Cnd* on *B_Rep_* calculated from *Z_cur_, Z_rec_*, and *Z_com_* seems to make sense when BACON is in-essence making a statistical inference as to its whereabouts. However, if a representation for the current context has just been created, BACON can in-effect be *certain* that its active new representation is valid. Thus, it might plausibly be argued that once the representation is created, conditionability should jump to unity for the remainder of that session, as indicated by the dashed step function in Figure 6A. Alternatively, one might argue, somewhat as in the previous paragraph, that for a given amount of information available about the current context, *Cnd* should not go *down* in a later session with the same information. In that case, the upper limit for *Cnd* on the session of a representation's creation would be the value it would be expected to have given random sampling of attributes on a later session. The bold red dotted curve of Figure 6A plots this upper limit.

Whatever the cogency of either argument, it appears from available data that in a novel context conditionability rises *gradually from zero* following the period during which no conditioning is possible (Fanselow, 1990), exactly as shown in the bold dashed curve of Figure 6A. We therefore have constructed BACON so that it computes *Cnd* in this way on the session of encoding.

#### Fear expression

Just as it would make little sense for fear to become conditionable before an animal has enough information about a context to know where it is, so too expression of previously conditioned fear should depend on an animal's confidence about its locale. We thus let the *effective* conductance (*Ge'*) produced by a potentiated excitatory CA3′-amygdala' synapse be dependent on *B_Rep_*, being zero when *B*_Rep_ = 0 and becoming equal to its full potentiated value *G_e_* when *B_Rep_* = *B_add_* (Figure 5) Specifically,

(6) $${G^{\prime }}_{e}=linsig(B_{Rep}|0,B_{add})\;▪\;G_{e}.$$

*G_e_'* is here taken to begin its increase at *B_Rep_*= 0, rather than at *B_old_* (compare to equation 4) because we suppose that it is better to err on the side of being afraid. The actual expression of fear is proportional to the depolarization *V_Amyg_* that is produced by the conductance *G_e_'*:

(7) $$V_{Amyg}={G^{\prime }}_{e}/(1+{G^{\prime }}_{e})$$

(see Methods). Thus, the onset of fear expression increases as *B_Rep_* rises during a session (Figure 6B).

During a session where representation creation occurred, *B_Rep_* in Equation (6) is replaced by *Expected B_Rep_*. This is used in computing post-shock freezing.

#### Neural implementation of control rules and bayesian calculations

Although the above logical description of the BACON algorithm fully characterizes its operation (and provides a sufficient basis for reading later sections), it is important to make clear that the operations specified above could in fact be carried out by neurons. There would be many ways of doing this; we sketch one here.

Central to this model is the control of plasticity and other neural properties. Since such control applies to entire classes of neurons and synapses, we think of it as being exercised via widely distributing neuromodulators. We assume that Recall mode is the default and imagine separate modulators for casting the network into Create and Update modes. We also employ a third modulator to signal the extent of positivity of *B_Rep_* (*posB*)for controlling *Cnd* and fear expression. Control of plasticity is assumed to be via direct effects on synaptic properties, though one could imagine the possibility of indirect control via modulation of firing rates. We note that the work of Hasselmo (2006) provides a precedent for supposing hippocampus to be thrown into different, neuromodulator-controlled modes of operation during encoding and recall and that a variety of neuromodulators are known to affect LTP (e.g., Frey et al., 1989; Markram and Segal, 1990; Huerta and Lisman, 1995; Thomas et al., 1996. Reviews: Kenney and Gould, 2008; Lovinger, 2010; Lisman et al., 2011; Hawkins, 2013).

**Figure 7:**
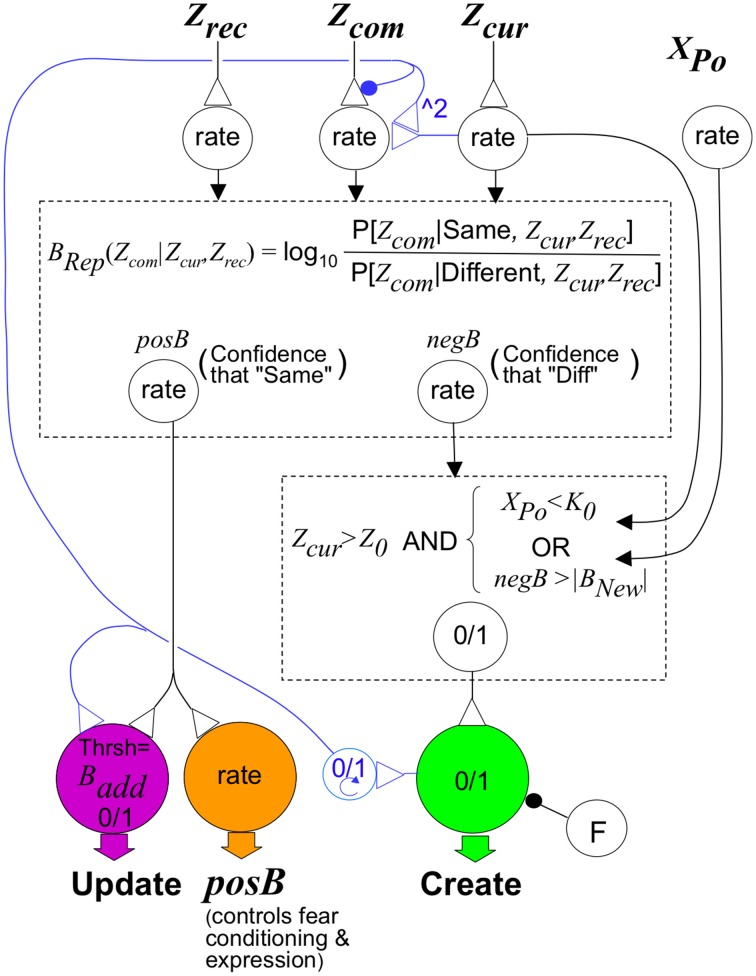
**Sketch of circuit that would implement BACON control rules**. The upper dashed box represents a circuit that, like that in Figure 3B, can compute *B_Rep_* in response to input from neurons whose rate of firing signals *Z_cur_, Z_rec_*, and *Z_com_*. It has as output, neurons that separately convey positive and negative values of *B_Rep_*. These, along with activity of a neuron whose activity level *X_Po_* is proportional to the number of neurons active in the pre-recurrent input activity pattern of CA3′ (_CA3_Ptrn_o_), determine the activity of the neuromodulator-releasing neurons shown at the bottom of the figure. The neuromodulator that configures the cortical-hippocampal circuit for representation creation is released when the logical conditions in the lower dashed box are met and the creation neuron is not inhibited by neuron F. We have written the logical conditions themselves rather than a neural circuit that would implement them because this makes the logic clearer, and there are many straight-forward ways to implement these rules neurally. Inhibitory input to the Create neuron from neuron F suppresses its activity if a representation (different from the current one) for which *B_Rep_ > B_pv_* was active earlier in the session (as explained in the section on creation and updating). Circuitry for controlling F is provided in the Supplementary Material (Topic D). The neuromodulator that configures the cortical-hippocampal circuit for updating is released when *posB>B_add_*. However, if a new representation has been created, the neuromodulator must continue to be released until the automaton leaves the context. This is mediated by a binary (“0/1”) working memory neuron that becomes active when representation creation occurs and remains so until BACON leaves the context. The binary neuron also reconfigures input to a neuron innervated by *Z_com_* so that (as discussed in the text section on Conditionability) it will reflect *Expected Z_com_* instead of *Z_com_* itself. This is done by inhibiting transmitter release from the *Z_com_* neuron and activating an input from *Z_cur_* that causes the target cell to respond as the square of *Z_cur_*; this in-effect computes the expected value of *Z_com_* given that *Z_rec_* equals *Z_cur_* when the automaton is updating representations. Control of fear conditioning and expression is mediated by a neuromodulator that is released in proportion to the *posB* signal. Its effect on conditioning and fear expression are as described in the text.

Figures 3B_1_,B_2_ sketch circuitry that can compute approximate Bayesian weights of evidence; as seen in Figure 3C, it does this fairly accurately. Figure 7 sketches circuitry that could implement the control rules described in previous sections. We think of prefrontal cortex as a plausible locus for this speculative control circuitry.

### Properties and performance of BACON

Conditionability and fear expression will vary over time, dependent on what attributes are randomly sampled. In order to avoid proposing a theory of sampling over time, which is a complex topic of its own, we use sample number (*Z_cur_*), rather than time, as an independent variable. However, a few comments about time *per se* are needed: The earliest that a representation can be created and conditioning occur is at sample *Z_0_*, and the earliest time that conditioning begins in rats is about 30 s (Fanselow, 1990). We therefore imagine that at the start of a session about *Z_0_* (=45) attributes are sampled per 30 s. However, we presume that any plausible theory of sampling would have sampling slow greatly from this rate as the proportion of total attributes sampled approached the total available. Indeed, we imagine that it would be rare for full knowledge of a context's attributes to ever be achieved.

It should be noted that BACON has intentionally not been designed to extinguish. Since extinction of context fear is time-dependent, extinction could not have been included without a model of sampling, which we wished to avoid. Moreover, the behavioral consequences of the model's Bayesian inference mechanisms are seen far more clearly in the absence of extinction. The approach proposed for adding extinction is sketched in the Discussion.

#### Illustrative simulations

**Figure 8:**
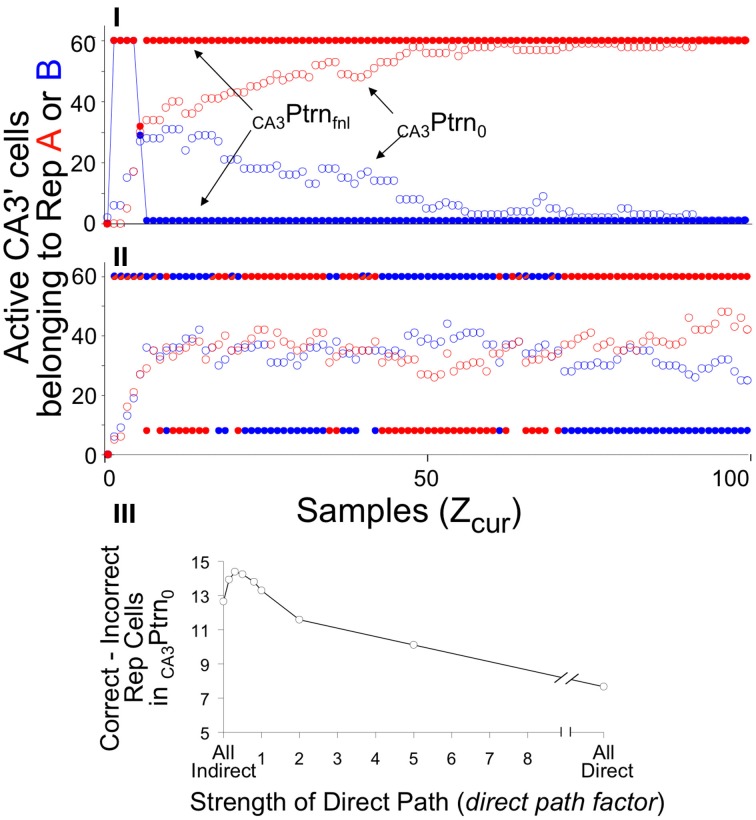
**_CA3_Ptrn_o_ and _CA3_Ptrn_Fnl_ activity during recall of context A when BACON is familiar with both it and another context B**. *Z_rec_* for A is 95 and for B is 80. **(I)** Contexts A and B are maximally distinct (50% of attributes in common—only those common to all contexts). **(II)** Contexts A and B are very similar (95% of attributes in common). In both cases a mix of Representation A and B cells is active in the initial CA3′ pattern prior to recurrent input (_CA3_Ptrn_o_), whereas whichever representation has the larger number of representatives active initially has all *K* = 60 of its members active after two cycles of recurrent input (_CA3_Ptrn_Fnl_). Note that since there is some overlap between the representations, the final pattern necessarily has some representation A cells active when representation B is being expressed, and conversely. When the contexts are very different **(I)**, the final pattern is consistently the complete representation of A after sampling only a few attributes, and by the time about half of the full set of attributes have been sampled, the initial pattern is also the complete correct representation. However, when the contexts are very similar **(II)**, the initial pattern is never either representation in pure form, and only after about two thirds of the total set of attributes has been sampled is the final pattern consistently the complete valid set of representation cells. Note that in **(II)** there were a number of occasions when the recurrent collateral system caused exactly equal excitation of both representations. Such cases are indicated by half red/half blue _CA3_Ptrn_Fnl_ points; there are therefore more than *K* neurons active in the final CA3′ pattern, and under that circumstance, EC'_out_ activity is suppressed. **(III)** The difference between the number of A and B representation cells in _CA3_Ptrn_o_ as a function of strength of direct path synapses (*dpf* = factor multiplying strength of direct path synapses on CA3′ cells), with patterns A and B 93% similar. This is an average of 24 sets of simulations. For each set the same random connectivity and the same attribute sampling orders were used for a simulation at each dpf value. Paired *t*-test for All Indirect vs. All Direct, All Indirect vs. *dpf* = 0.15, and *dpf* = 0.3 vs. 1.0 were all significant at *p* < 0.01 or better.

Figure 8 illustrates the operation of BACON's hippocampus in recall mode. For this simulation the automaton was first given sufficient exposure to two contexts (A and B) to cause creation of representations for each. Figures 8I,II show activity during recall tests in context A. In Figure 8I context A and B were fairly distinct, whereas in Figure 8II they were very similar. It should be borne in mind that the automaton's knowledge of those contexts for which it has representations will usually, as here, be incomplete unless it has spent a great deal of time in those contexts. Moreover, because of the vicissitudes of random attribute sampling, attributes common to two different contexts will often have become associated with one representation but not the other. Thus, if tested in either context, where it will again sample attributes at random, it may well sample common attributes that by chance became associated with the other context, and early in a session these might by chance predominate. As a result, EC'_in_ activity will often activate some elements of incorrect representations in CA3′. The graphs show the number of active CA3′ units that were part of the representations of each context as sampling progressed during the test. This is shown both prior to input from the recurrent collateral system (_CA3_Ptrn_o_) and after the final iteration of such input (_CA3_Ptrn_Fnl_). In both simulations there was a mix of elements of each representation active prior to recurrent collateral input, but in the post-recurrent input representations, whichever pattern had the most active elements prior to recurrent input prevailed. Moreover, as sampling progressed, the valid representation increasingly predominated. When A and B were quite different, the final output of CA3′ was the valid representation starting fairly early in the session; when they were quite similar, it took many samples before the final representation was consistently the valid one.

This sort of simulation provides a good opportunity to evaluate the consequences of using the direct vs. indirect pathways during recall. As will be explained in Discussion, it was expected that use of the indirect path alone during recall should lead to more reliable activation of the actual current context's representation than use of only the direct one. However, both operating together should produce somewhat better performance than either operating alone. The ratio of strength of direct path to indirect path synapses in recall mode is the parameter *dpf*. In simulations I and II only the indirect pathway was operational (*dpf* = 0). Figure 8III shows the preponderance of Representation A over B neurons in CA3′ prior to recurrent collateral input, as a function of *dpf* in simulations like those in I and II. As expected, the indirect pathway produces better separation than the indirect one, but both together are best. However, the advantage of incorporating input from the direct pathway is modest and is seen only if input from the direct pathway is relatively weak. For simplicity's sake we chose to use only the indirect pathway (i.e., *dpf* = 0) in the simulations below.

**Figure 9:**
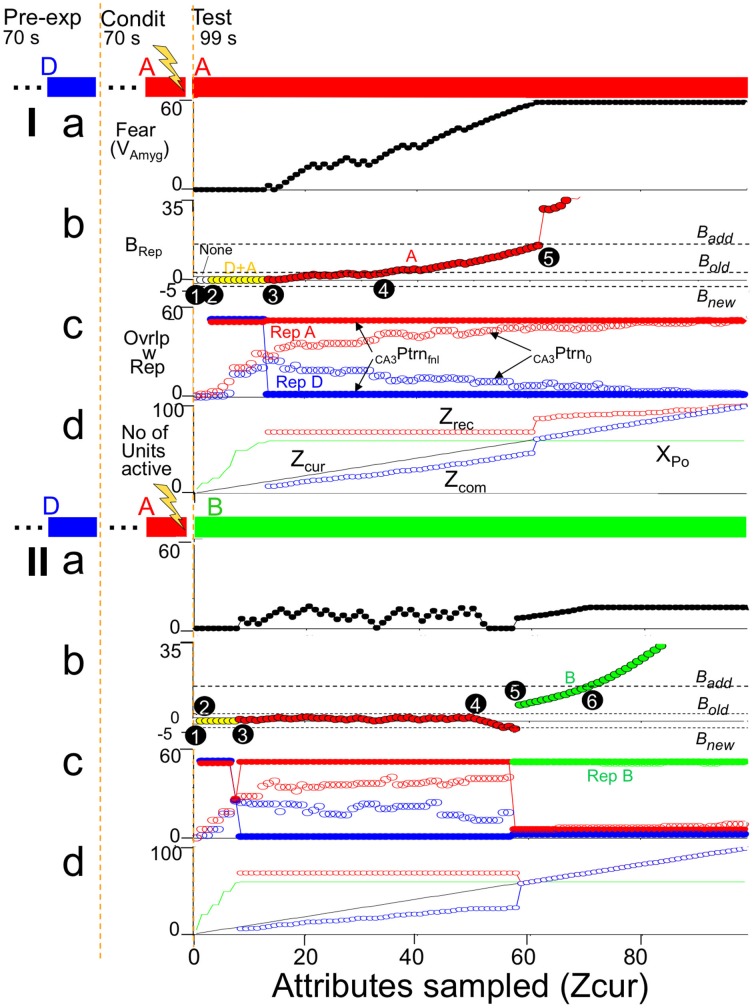
**Illustrative simulations**. BACON was pre-exposed to context D (blue—70 samples) and then given a US at the end of 70 samples in a novel context A. Context A was maximally different from D (the only attributes common to them were the 50 attributes that were part of all contexts). A subsequent test session (which is what is shown in detail) was either **(I)** in context A or **(II)** in a partially similar context B (green—75 attributes common to A). The bars at the top of the figure indicate what context the automaton was in throughout the simulation. **(a)** Fear. **(b)** *B_Rep_*. The active representation that was controlling EC'_out_ activity and determining *B_Rep_* is indicated by marker color. **(c)** Overlap of active _CA3_Ptrn_o_ and _CA3_Ptrn_Fnl_ cells with each existing representation. **(d)** *Z_cur_, Z_rec_, Z_com_*, and *X_Po_* (i.e., number of cells active in _CA3_Ptrn_o_) as a function of sample number. Details of black numbered points: **(I)**. **(1)** For the first several samples the number of cells active in CA3′ prior to recurrent input (*X_Po_*) was less than the *K_0_* neurons needed to produce further CA3′ activity, so in **(c)** there is no _CA3_Ptrn_Fnl_. **(2)** After a few samples, this ceased to be the case, but attributes that were common to all contexts were sampled in such a way that _CA3_Ptrn_Fnl_ included both A and D representations; therefore it included more than *K* active neurons, and EC'_out_ activity was suppressed (no *Z_rec_* and *Z_com_* in **d** and *B_Rep_* = 0 in **b**). Although some of the CA3′ neurons now active made potentiated synapses with amyg' neurons, no fear is expressed because *B_Rep_* = 0. **(3)** By this time, enough attributes unique to context A had been sampled so that _CA3_Ptrn_Fnl_ was the complete context A representation. CA3′ therefore drove the EC'_out_ cells with which the CA3′ cells had potentiated synapses, *Z_rec_* became 70, and *Z_cur_* and *Z_com_* were sufficient to generate a slightly positive *B_Rep_*. This allowed some transmission at active, potentiated CA3′-amyg' synapses, which caused contextual fear expression to begin. As *Z_cur_* and *Z_rec_* further increased, *B_Rep_* grew and produced more fear. **(4)** *B_Rep_* reaches *B_old_*. Conditioning therefore became possible (i.e., *Cnd* (not shown) went positive). **(5)** At sample 62 *B_Rep_* reached *B_add_*. From then on there was full expression of the fear that had previously been conditioned. Moreover, sampled attributes that had not previously become associated with the context could now become so. As a result there was a sudden increase in *Z_rec_* and thereafter a further increase whenever another not already known attribute was sampled. As *Z_cur_*, *Z_rec_*, and *Z_com_* increased, so too did *B_Rep_*. **(II). (1)** Initially *X_Po_* was < *K_0_*, so there was no _CA3_Ptrn_o_ activity. **(2)** Both RepresentationA and B cells were active in _CA3_Ptrn_Fnl_; thus *Z_rec_*, *Z_com_*, and *B_Rep_* are zero. **(3)** As more attributes were sampled, the representation of cntxt A, the conditioning context, which was more similar to the current context than to cntxt D became expressed in _CA3_Ptrn_Fnl_. During some of this time, there was a small positive weight of evidence (*B_Rep_*) for the validity of this representation and a little fear was expressed. **(4)** As the sampling continued, attributes that were not among those associated with the currently active conditioning context were sampled and provided evidence that the current context was not B; this caused a negative *B_Rep_* value to emerge. When *B_Rep_* was negative, no fear was expressed even though the hippocampal representation of the conditioning context was active. **(5)** At sample 57 *B_Rep_* fell below *B_new_*, and since *Z_cur_* was greater than *Z_0_* (=45), a representation of the current context (context B) was created. Because the representations of A and B had a few cells in common, there is some fear expressed, which grew as the *B_Rep_* value for context B's representation increased. **(6)** *B_Rep_* reached *B_add_*. Increases in *B_Rep_* beyond *B_add_* do not cause any increased expression of whatever fear has been conditioned (Equation 6), so fear levels off. Fear leveled off when BRep reached Badd, because could not increase further once because *B_Rep_* reached *B_add_*.

Having considered the activity of the hippocampus alone, Figure 9 brings in the rest of the circuit. It shows two simulations. In both, BACON was fear-conditioned in a context A after pre-exposure to a very different context D. It was then tested either in A (Figure 9I) or in a different but somewhat similar new context B (Figure 9II). The colored bars at the top of Figures 9I,II show what contexts the automaton was in and when shock was given. Note first that in Figure 9Ic, as in Figure 8I, when BACON was tested in a familiar context after having been exposed to it and another fairly dissimilar one, CA3′ activity separates well even prior to recurrent collateral input, and _CA3_Ptrn_Fnl_ becomes the complete representation of context A early in the session. On the other hand, when placed in a different unfamiliar context B that was moderately similar to A, the hippocampus initially activated the representation of A, but eventually (after having acquired enough information about the current context to show that A is invalid) created a new representation for B.

Figure 9Ib plots *B_Rep_* for the active representation throughout the simulation, with which representation was active indicated by point color. When in context A, the representation of A became the sole representation active at sample 15 (black circle point 3). This allowed EC'_out_ neurons to be activated (*Z_rec_*~70, Figure 9Id). Since most of the currently sampled attributes overlapped those then active in EC'_out_ (*Z_com_* slightly less than *Z_cur_* in Figure 9Id), *B_Rep_* took on a positive value, and since the context A CA3′ representation cells now active were those to which fear was conditioned, fear expression began to rise (Figure 9Ia). As more attributes of A were sampled, *Z_cur_* and *Z_com_* rose (Figure 9Id), and so *B_Rep_* and fear increased correspondingly (Figure 9Ia). At black circle point 5, *B_Rep_* reached *B_add_*. This allows maximal expression of conditioned fear and allows updating of the representation of A so all sampled attributes that were not already associated with the representation of A became so causing sudden increments in *Z_rec_* and *Z_com_* (Figure 9Id).

When placed in context B (unfamiliar, but moderately similar to A), the representation of A was activated early on (Figure 9IIc). The attributes of A and B were sufficiently similar so that a positive *B_Rep_* value developed at about sample 8 (Figure 9IIb), with a corresponding modest expression of conditioned fear (Figure 9IIa), but by sample 60 (black circled point 5) differences between current attributes and the recalled attributes of representation A became so great (Figure 9IId) that *B_Rep_* fell below *B_new_* (point 5 in Figure 9IIb), and a representation for context B was created. Once that happened fear expression occurred only because the representation of context B had some neurons in common with that of context A.

#### Representation characteristics

Representations in BACON, even of quite similar contexts, have only limited overlap (i.e., the encoding (creation) process produces considerable “pattern separation”). Two factors contribute to the slightness of overlap. First, as often discussed, the large number of DG' neurons, their innervation by random groups of EC_in_ cells, and the firing of only the *K* most excited, promote pattern separation; these factors cause contexts with 90% overlap at the level of EC_in_ to have about 30% overlap at DG' during representation creation. Second, contexts are generally created when only a fraction of their total attributes have been (randomly) sampled. With this factor operating along with the first, overlap of the representations for 90% similar contexts falls to about 10%. As a result, even when two contexts are extremely similar, relatively distinct sets of attributes will control the form of their representations and very different representations result.

#### Conditionability—the immediate shock deficit and pre-familiarization

When placed in a novel context that is fairly distinct from any familiar one, a representation of the context will be created at sample *Z_0_*. Conditionability begins to increase at this point and continues to do so as *Z_cur_* increases, until it reaches its maximum value of unity (Figure 6B, bold red dashed curve). This delay, with subsequent gradual increase in conditionability, corresponds to the immediate shock deficit of real context fear conditioning (Figure 6C).

If placed in a *familiar* context (i.e., one for which a representation was previously established) conditionability begins to rise when *B_Rep_* reaches *B_old_*. The more that is known about the context's attributes (i.e., the greater *Z_rec_*) at the start of the session, the sooner *B_old_* will be reached (Figures 4A, **6A**). Figure 6D illustrates such an earlier rise in conditionability following pre-familiarization with the to-be-conditioned context in a real experiment on the immediate sock deficit. In the theoretical case the rate of rise is generally greatest in the session of creation, because expected conditionability is proportional to *Expected B_Rep_* which is an increasing function of both *Z_cur_* and *Z_rec_*, and whereas *Z_rec_* increases at each successive sample during the session of encoding, on later sessions it is constant throughout a session unless *B_Rep_* comes to exceed *B_add_*.

It is a basic feature of BACON that the attributes of a novel context cannot begin to be learned until a hippocampal representation is formed, and this cannot occur until the number of samples made exceeds *Z_0_*. Therefore, even many repeated exposures to the context, each of which is too short for *Z_0_* samples to be made, will not reduce the immediate sock deficit. Moreover, attributes cannot be added to an existing representation unless *B_Rep_* exceeds *B_add_*. Therefore, once BACON has been familiarized sufficiently with a novel context to allow some measure of conditioning, further later exposures will only be effective in shortening the immediate shock deficit if some of the exposures are long enough to allow *B_Rep_* to exceed *B_add_*. To our knowledge no experiments that would evaluate either of these features have been published.

#### Fear expression—delayed onset of fear and effects of context familiarity

Fear expression in BACON develops gradually when the automaton is introduced into to a feared context (Figure 6B). As illustrated in Figure 6E, fear-induced freezing behavior does in fact develop gradually, though because extinction sets in, the rise is followed by a decline that is not emulated by BACON because we chose not to incorporate extinction mechanisms.

Since both conditionability and fear expression become maximal when *B_Rep_* reaches *B_add_*, both become maximal at about the same time (Figures 6A,B—note dashed lines between frames Figures 6A,B).

#### Generalization

In BACON, fear conditioned in one context may generalize to another similar context for either of two reasons: (1) The hippocampal representations of the two contexts may have neurons in common. (2) The vicissitudes of attribute sampling may sometimes cause periods of activation of the hippocampal representation of a familiar context *similar* to the actual context rather than of the actual context itself. The latter is almost certain to occur if the context does not yet have a representation of its own. We chose the automaton's parameters so that for contexts with 90% attribute overlap, hippocampal representations would only overlap by about 10%. Therefore, generalization in BACON is usually attributable more to misidentification of context than to representation overlap.

**Figure 10:**
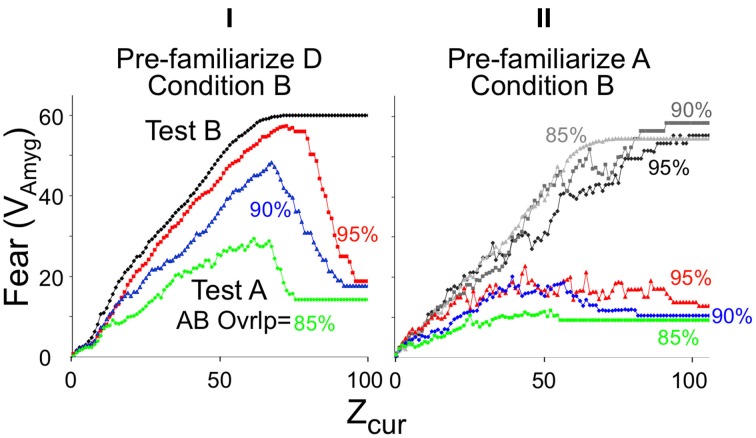
**Generalization of context fear**. BACON was conditioned by a shock at the end of a session in novel context B and then tested either in the conditioning context (black and gray curves) or in a generalization context A (colored curves). Context A's similarity to B was either 85% (green), 90% (blue), or 95% (red). The automaton was pre-exposed to either the generalization context (A) **(II)** or to a very different context (D) as a control for per-exposure *per se* **(I)**. Because degree of conditioning is somewhat affected by pre-exposure to a context similar to the conditioning context, separate conditioning-context tests had to be run for each pre-exposed generalization context (shades of gray in **II**). Each curve is the average of 30 simulations. The pre-familiarization session was 95 samples, the conditioning session 80, and the test session 99.

Degree of context fear generalization is very dependent on the extent to which the automaton is already familiar with the context in which generalization is tested. Figure 10I illustrates generalization when BACON is not familiar with any contexts that are at all similar to that in which it has been conditioned. During generalization tests in such cases there is initially substantial generalization of fear because the automaton at first misidentifies a similar but unfamiliar test context as the conditioning context itself. But once it is recognized that the test context is a new one, a representation for it is created, and fear then falls off; the only fear that then remains is that caused by those representation neurons that are common to the test and conditioned context. However, if BACON was pre-familiarized with the test context (Figure 10II), only a slight initial surge of fear occurs, because the pre-established representation of the test context itself quite rapidly becomes active.

Pre-exposure to the conditioning, as opposed to the test context also diminishes generalization (not illustrated). This happens because the better-known the conditioning context (the greater its *Z_rec_*), the more rapidly a similar context will be recognized as different. Decreased generalization of stimuli that are better known has been observed both for context fear and other forms of conditioning (Kiernan and Westbrook, 1993; Rudy and O'Reilly, 1999).

#### Effects on conditioning of familiarity with similar contexts

**Figure 11:**
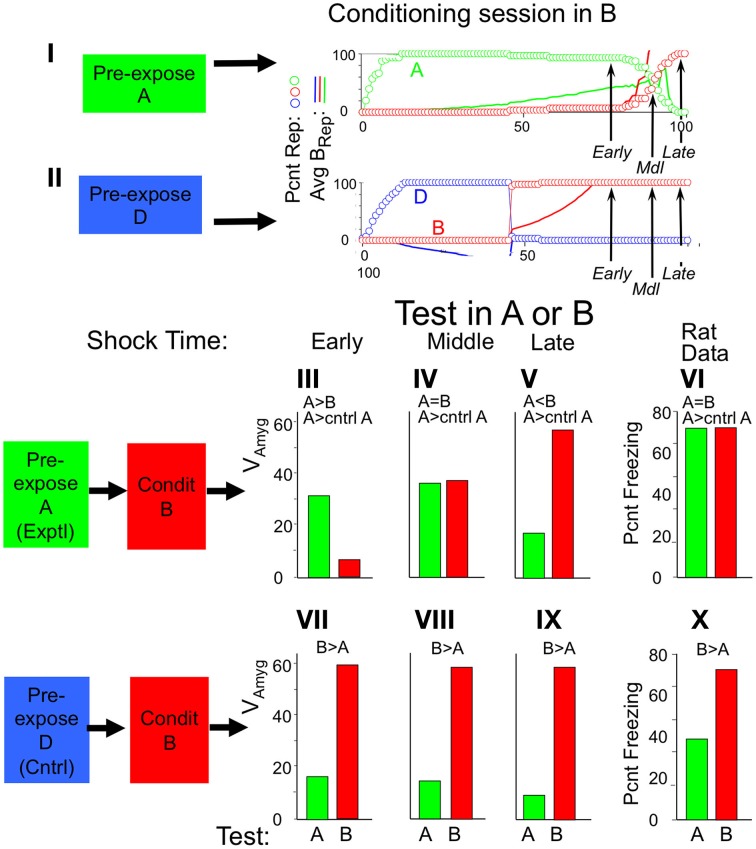
**Conditioning in a novel context when a similar context is familiar**. Context A and B are 95% similar; context D is maximally different from both (50% similar). **(I,II)**. Sessions in context B after pre-exposure to either context A or D. Arrows (*Early, Middle, Late*) indicate when USs were given during each type of conditioning session. Circles plot percentage of runs in which the specified representation was active, and lines the mean weight of evidence for the representation (same color coding). **(III–X)** Average fear levels in contexts A (green bars) and B (red bars) after conditioning in B following pre-exposure to either context A or D. First 3 graphs in each row are simulation results for test following conditioning with USs at the early, middle, or late time points. Pre-exposures were always 60 samples; conditioning sessions were 75 (*Early*), 88 (*Middle*) or 95 (*Late*) sample long with US at end. All figures are averages for 30 simulations. **(VI,X)**. Far-right graphs are Rudy and O'Reilly data (Rudy and O'Reilly, 1999).

Rudy and O'Reilly (1999; see also Rudy et al., 2002) have published intriguing experiments in which, when rats were shocked in an unfamiliar context similar to one they knew, fear became conditioned to the familiar but absent context. BACON does something similar. The reason can be seen in Figure 11I, which shows what representations are active when BACON is placed in an unfamiliar context B similar to a context A to which it was pre-exposed. Initially the existing representation of A is activated because B has many of the same attributes as A. As sampling progresses, the automaton becomes more and more “certain” that it really is in A, as reflected by the gradual increase of A's *B_Rep_* value (and as discussed above with respect to Figure 4B). But eventually sufficient discrepancies between currently observed attributes and those associated with A's representation mount to a point where a representation for B itself is created (and its *B_Rep_* value then increases rapidly). If a US were given at the point marked “Early” fear would be conditioned to context A, if given at the “Middle” point, each representation would be active in about half the runs, and if given “Late,” conditioning would be to context B.

Had BACON been pre-familiarized with a context, D, very different from B, conditioning at the corresponding points in the experiment would always have been to the actual context, B (Figure 11II).

Fear expressed in each context after giving BACON USs at one of these three points are shown in Figures 11III–V, VII–IX. When BACON had not been pre-exposed to A, it always showed considerable fear of B but little of A, though some generalization did occur. However, when it had been pre-exposed to A, the outcome depended on when the US was given (Figures 11III–V). When given Early, fear of A was greater than B (Figure 11III) (and also greater than fear of A in the absence of pre-exposure to it). When given Late, fear during testing was greater for B (the actual conditioning context) than for A (Figure 11V). When the US had been at the Middle time point, fear was expressed mostly to A in some runs and to B in others (not shown), with the average across simulations being about equal for both (Figure 11IV). In the actual experiments (Figures 11VI,X) the pattern of results is similar to those of the Middle time point simulations. In the experiments of panels Figures 11VI,X two shocks were given, one at 120 and another at 240 s. In that case one could imagine the first being at our “Early” and the second at our “Late” time point. However, in other experiments, which had similar results, a single shock was given, as in our simulations.

## Discussion

### Multiple modes of hippocampal function—necessity and benefits

The algorithm described here was an attempt to construct a neurally plausible mechanism for context fear conditioning. The role of the hippocampus in this is the creation and subsequent recall of permanent, relatively non-overlapping representations of contexts to which associations can be made. We assume that at each entry to a context its attributes are discovered serially in random order. Since attributes of a context will be sampled in different orders on different occasions, and since information about a context will usually be incomplete when its representation is created, determining whether a context is novel (in which case a representation for it should be created) or familiar (in which case pattern completion mechanisms should be allowed to operate) is a challenging problem. This led us to construct for BACON extra-hippocampal machinery designed to make optimal decisions as to the novelty or familiarity of whatever context it was in, and then to allow this machinery to configure the hippocampus specifically for representation creation, updating, or recall, as required.

This strategy solves two problems that arise if the hippocampus is assumed to have only one state:

1. In conventional theories of the hippocampus the occurrence of a novel stimulus configuration that is similar to one previously encoded may activate the complete code of the familiar stimulus with the result that a new representation will not be created but its attributes will, improperly, become associated with the existing representation. Or conversely, in the presence of a known stimulus, incomplete sampling of its attributes or sampling of attributes not originally encoded will cause a new representation for it to be created so that one stimulus will now have two different (pattern-separated) representations. This is the so-called “pattern separation-completion tradeoff” that has been extensively discussed in the literature (O'Reilly and McClelland, 1994; and see O'Reilly and Rudy, 2001). The sort of control of hippocampal mode that we have utilized in BACON essentially abolishes this problem, because decisions as to novelty vs. familiarity are made optimally, and hippocampal plasticity is shut off when it would be inappropriate.
2. Although, as will be explained below, pattern recall should work best if information about a stimulus reaches CA3′ via the indirect (EC'_in_-DG'-CA3′) pathway, it has been argued, and is often accepted, that properties of the indirect pathway that are needed for optimal representation encoding make it impossible for the pathway to also be used effectively during recall (Treves and Rolls, 1992; O'Reilly and McClelland, 1994; Treves et al., 2008). However, this sort of argument loses all force if, as is done in BACON, the properties of the hippocampus can be configured for its different modes of operation.

### DG-CA3 projection

In BACON each DG' neuron has a single dedicated CA3′ partner with which it makes a non-plastic synapse. In the actual hippocampus there are less CA3 than dentate cells, and each dentate cell innervates a small number of CA3 cells, via synapses that show some sort of plasticity, but not the type of associative NMDA-dependent plasticity seen at EC-dentate and recurrent CA3 network synapses (Bortolotto et al., 2003; Reid et al., 2004). In so far as a KWTA competition within DG' is used to choose the cells for a representation, it seemed to us to make good design sense to let those cells have specific partners in CA3′. Thus, representational elements are in-effect DG'-CA3′ dyads that, during recall, are excited by potentiated inputs from EC'_in_ and that are bound to other dyads of the same contextual representation by the potentiated synapses of the recurrent collateral network.

CA3′ cells could have been paired with DG' cells by making the synapse between a DG' cell and one of a small number of CA3′ cells it innervates functional only if and when the DG' neuron won a KWTA competition during representation creation. Something like this seems to happen during real nervous system development where activity-dependent mechanisms cause vertebrate muscle fibers, cerebellar Purkinje cell, and thalamic neurons to become innervated by single motor neurons, climbing fibers, or topographically close thalamic afferents (e.g., Favero et al., 2009; Kano and Hashimoto, 2009). However, for us it was simplest to just take the existence of DG'-CA3′ pairs as given. Nevertheless, it should be noted that a given DG'-CA3′ pair will never get used if the sets of attributes whose EC' representations innervate the DG' cell never occur together in the “life” of the automaton. Therefore, pairing up a CA3′ cell with an active DG' cell at the time of representation creation would have allowed a smaller CA3′ layer and would have reduced the size of the huge recurrent collateral network. One can imagine that this could be happening in the biological hippocampus and could account for the smaller number of CA3 than dentate neurons, the limited fan-out of dentate neurons, and the presence of atypical plasticity at dentate-CA3 synapses.

### Direct/indirect pathway balance in recall

Since, as discussed above, we can ignore computational arguments against a role for the indirect pathway during recall, we consider the relative merits of using it vs. the direct one: Each DG' and CA3′ cell will be innervated by *F* = 60 of the *N*_Ctx_ = 1000 EC' attribute cells. The innervation of each DG' and CA3′ cell is chosen *randomly* and *independently*; thus the two members of a DG'-CA3′ pair do not receive input from the same set of EC' attribute cells. When the representation for a context is created, there will usually be on the order of *Z*_0_ = 45 EC'_in_ attribute cells active, and on average each DG' and each CA3′ cell will be innervated by (a different) 2–3 of them. The synapses of the active attribute cells on the *K* DG' and CA3′ cells of the forming representation will become potentiated. In CA3′ it will be the approximately 2–3 active EC'_in_-CA3′ synapses that innervate each cell that become potentiated. However, in DG' the cells that are active will be *selected* as the *K* that are most excited and therefore most heavily innervated by the active EC' attribute cells. The DG' cells that are active will therefore have a richer active EC' attribute cell innervation than will the population as a whole, and it will be some 7–8 synapses on them that will become potentiated.

During recall, DG' and CA3′ cells receive information from EC'_in_ only via potentiated synapses. In order for a representation cell for a context *A* to be selectively activated by context *A*, as opposed to a different but similar context *B*, a representation cell for *A* must receive effective input from attribute-representing neurons that are specific to *A*, and if *A* and *B* are very similar, there will not be many *A*-specific attribute neurons. So when very similar contexts must be differentiated, the DG' cells, which get effective input from 7 to 8 EC'_in_ cells, are more likely to get input from a given attribute (which will be passed to a cells CA3′ partner) than will the CA3′ cells, which get effective input from only 2 to 3 EC'_in_ cells. Thus, if only the direct or only the indirect pathway were used for recall, the indirect would be better because it would be more likely to provide effective input from a context *A*-specific neuron. However, if both the direct *and* the indirect pathway provide information that determines the response of CA3′ cells prior to recurrent input, differentiation should be somewhat better than with either pathway alone. As shown in Figure 8III, the effective separation of the representations of two similar contexts at CA3′ prior to recurrent input (i.e., _CA3_Ptrn_o_) as a function of the relative strength of the direct pathway is in fact just as expected. Moreover, it seems to be optimal for the direct pathway to be weak relative to the indirect one. This may be seen as consistent with the biological situation, where both pathways are present, but the direct one makes relatively distal synapses on CA3 neurons.

### From CA3′ to EC'_out_

As explained at the start of Results, we have allowed CA3′ to innervate EC'_out_ directly, rather than via a CA1-like processing stage because complete innervation in the recurrent collateral network and in EC'_out_ made a CA1′ redundant. Since it is the Shaffer collateral synapses of CA3 cells on CA1 neurons that have told us much of what we know about Hebbian LTP, it may seem ironic that we did not explicitly include a CA1 in the algorithm. However, in the current model, the Hebbian nature of *EC synapses on dentate and CA3 cells* are sufficient to produce effective encoding and recall of contextual representations. In a model of this type CA1 plasticity would serve mainly to help mitigate any errors or omissions that remained in representations after their processing by the recurrent collateral network.

### Functioning of DG' and CA3′ during representation creation and recall

There have been a number of experiments in which activity in dentate or CA3 in novel or familiar contexts has been assessed or manipulated. We consider here how BACON would behave in some of these.

#### Activity in novel contexts

BACON will start its time in a novel context in Recall mode, but fairly soon *B_Rep_* will fall below *B_new_* and a new representation will be created. For the remainder of the session, as each additional attribute gets sampled, excitation will be passed to CA3′ over the direct and indirect pathways, _CA3_Ptrn_Fnl_ determined, and since the system is in Update mode, _CA3_Ptrn_Fnl_ will be reflected back to DG' so that the synapses between the added EC'_in_ attribute cells and DG' cells can potentiate. From then on in each computational cycle hippocampal activity will consist of the *K* DG'-CA3′ pairs of cells of the current representation fully active, with the rest of the cells of each layer silent. Moreover, these active cells will overlap relatively little with those of other representations. Consistent with this, it does appear that, whereas there is a lot of overlap between the DG' cells active in different *familiar* contexts, during exposure to *novel* contexts DG' activity is quite context-specific, even when the contexts are similar (Deng et al., 2013) (Table 3, A).

**Table 3:** **Some behavioral properties of BACON**.

**Prediction**	**Evidence (for, against, nil)?**
A. In CA3′ only a small number (*K*) of cells will fire. During the first exposure to a novel context, there should be a fairly sudden shift in the population of active cells as a representation for the context is created, and the population of active cells should thereafter be constant. For familiar contexts, the same cells should always be active during different exposures to the same context, and substantially different cells active in different contexts. Even quite similar familiar contexts should activate fairly different populations of cells	For^a^^,^^b^
B. In DG' patterns of activity will be very different for the remainder of sessions in which new representations are created (i.e., in novel contexts) than at other times. Following representation creation activity patterns will have the same properties as those in CA3′ (few cells active; little overlap in cells active in different contexts). At other times cells will be active at graded rates, and firing rate profiles across cells will differ more, the more different the contexts	For^a^^,^^b^^,^^c^
C. Upon introduction to a distinctive *unfamiliar* context, context fear conditioning will be impossible until after a delay (we refer to the delay for an unfamiliar context as “D_0_,” which is about 30 s.), after which conditionability gradually increases (the immediate shock deficit)	For^d^^,^^e^^,^^f^
D. Upon introduction to a *familiar* context, there will also be a delay before context conditioning can occur, but it will be a time D that is shorter than Do	For^e^^,^^g^
E. Repeated exposures of duration less than the D_0_ to an unfamiliar context will not shorten D	Nil
F. No amount of pre-familiarization will completely abolish the immediate shock deficit (i.e., reduce D to zero)	For^e^ Against^g^^,^^h^
G. Upon introduction to a feared context, expression of fear will increase gradually	For^i^^,^^j^^,^^k^
H. Fear expression will rise gradually from very near the start of a testing session (in contrast to conditionability, which will begin to rise when *B_Rep_* reaches *B_old_*). However, conditionability and expression will reach their maxima at the same time (when *B_Rep_* reaches *B_add_*) and thereafter be constant	Nil
I. Generalization of fear *to* familiar contexts will be less than to unfamiliar ones	Nil
J. Generalization of fear *from* more familiar contexts will be less than from less familiar ones	For^h^^,^^l^
K. Conditioning to a novel context that is very similar to a familiar one may be to the familiar rather than the actual context	For^l^^,^^o^
L. The above effect will be less, the greater the delay of the US	Nil
M. Generalization tests will often show initial fear that later falls off much too fast to be explained by known rates of extinction	Nil
N. Loss of indirect path input to CA3′ during both encoding and recall does not seriously disrupt encoding or recall, but does reduce ability to distinguish similar contexts. However, inactivation of DG' during encoding alone has substantial effects on recall of contextual fear	For^m^^,^^p^
O. Inactivation of DG' plasticity has no effect on selection of cells during encoding, but reduces ability to distinguish similar contexts during recall, because without EC'-DG' plasticity recall must occur entirely via direct path	For^n^

a *Deng et al. (2013)*.

b *Leutgeb et al. (2007)*.

c *Neunuebel and Knierim (2014)*.

d *Fanselow (1986)*.

e *Fanselow (1990). Also Fanselow, unpublished*.

f *Blanchard et al. (1976)*.

g *Matus-Amat et al. (2004). During pre-exposures and conditioning, rats were consistently carried to the test chamber in the same covered black bucket; the black bucket could therefore be considered a part of the context, and “immediate” shocks should be seen as in-effect occurring at the transport- time after the beginning of contextual exposure*.

h *Kiernan and Westbrook (1993). Whether, as in the previous experiment, a consistent environment immediately preceding nominal context entry may have made the “immediate” shock in-effect somewhat delayed is not known*.

i *Wiltgen et al. (2006)*.

j *Fanselow (1982)*.

k *Lester and Fanselow (1986)*.

l *Rudy and O'Reilly (1999)*.

m *Nakashiba et al. (2012)*.

n *McHugh et al. (2007)*.

o *Rudy et al. (2002)*.

p Kheirbek et al. (2013).

#### Activity in familiar contexts

In a familiar context BACON will operate in Recall mode until such time as it “decides” it almost certainly “knows” where it is (i.e., *B_Rep_* > *B_add_*) at which time Update mode will begin to operate; we will assume for discussion that it is mainly Recall mode that will be observed during most experiments. During recall, it is DG's job to pass to CA3′ information as to how much excitation each DG' cell has received from EC'_in_; therefore, during recall the KWTA rule does not apply in DG', and cells there fire in proportion to their excitation. Thus, in DG' any cell that represents an encoded context having attributes in common with the current one will fire to some degree. However, in CA3′ the KWTA rule is operative. _CA3_Ptrn_o_ will typically include cells (possibly many but usually a minority) that do not represent the current context, because some representation cells of other previously encoded contexts may happen to have dense innervation from common attributes that happen to be in the current attribute sample. However, _CA3_Ptrn_Fnl_, once it emerges, will (if enough attributes have been sampled) be the complete representation of the current context and will overlap little with the representations of other, even similar, contexts. We assume for the sake of discussion that it would be mostly this final pattern of activity that would be detected in experiments. In fact, recordings from real animals do seem to show that in familiar contexts DG' uses a rate code with cells active in different contexts overlapping considerably, whereas in CA3′ there is little overlap between those active in different but similar contexts. Leutgeb et al. (2007), Neunuebel and Knierim (2014), Table 3, B; see Supplementary Material, Topic F for further discussion and a simulation of the Leutgeb experiment.

The proportion of sampled cells firing during tests in one of two familiar contexts in rat dentate has been reported to be about a third that in CA3 (Leutgeb et al., 2007). However, since rat dentate has about 8 times more cells than CA3 (O'Reilly and McClelland, 1994), this means that in absolute terms there are perhaps some 2.5 times more cells active in rat DG than CA3 during recall. In BACON each DG' cell innervates a single CA3′ cell, but whereas in Recall mode the number of cells active in CA3′ is limited to *K*, there is no limit in DG', and each cell there fires in proportion to its degree of excitation by sampled attributes that have potentiated synapses with it. In any given context, a given attribute would excite any cell it innervated that had previously become one of the representation cells of some context. All *K* DG' cells that had been active during the formation of the current representation would be excited plus presumably quite a number more. Thus, the observation that 2.5 times more cells are active in DG relative to CA3 in a familiar environment seems consistent with what BACON would predict (see also Supplementary Material Topic F).

#### Inactivation of dentate

Several studies have examined the effect of inactivating DG'. In one study, dentate-CA3′ transmission was suppressed with tetanus toxin during encoding, conditioning, and recall with only very modest effects. Fear expressed during a test in the conditioning context was roughly normal, as was degree of generalization to a quite different context, but there were some deficits in discrimination learning (Nakashiba et al., 2012). Apparently consistent with this, a more recent study found apparently normal fear during a test with DG' inactivated following encoding and conditioning with DG intact (Kheirbek et al., 2013). However, if DG was suppressed *during* encoding and conditioning but *not* during recall, fear of the conditioned context was diminished (Kheirbek et al., 2013).

BACON would emulate these observations under similar conditions: If DG' were inoperative during both encoding *and* recall, the indirect pathway would be used in both cases, and recall would be fairly normal, though discrimination of similar contexts would be somewhat reduced because one would lose the cooperative advantages of using both pathways, as discussed above. However, if DG' were suppressed only during encoding, there would be a great deficit in conditioned fear. The CA3′ cells selected to comprise the representation would be those most richly innervated by active EC'_in_ neurons via the *direct* path, and it would be only they that would become potentiated as part of the representation creation process. However, during recall with both pathways operative, input via the *indirect* path dominates. But because synapses to DG' did not become potentiated during encoding, the input to CA3′ that determines _CA3_Ptrn_o_ will be via DG' input synapses that had become potentiated during *previous* encodings when DG' was intact and will not promote the firing of representation neurons for the *current* context. If, on the other hand, DG' were suppressed only during recall, there would be only a modest effect, as seen in Figure 8III and as discussed in “Direct/Indirect pathway balance in recall,” above (Table 3, N). There would also be only a modest deficit if DG' plasticity were permanently eliminated: During encoding DG' would still select DG'-CA3′ dyads, but since potentiation would be impossible in DG', it would be EC'in-CA3′ synapses that would become potentiated and determine what CA3′ cells would be activated during recall. Consistent with findings of McHugh et al. (2007), there would be some deficits in discrimination ability but no major dysfunction.

#### Direct optogenetic activation of representation cells

Liu et al. (2012) recently studied mice in which dentate cells active during encoding of a context A were labeled with channelrhodopsin so that they could be selectively activated by stimulating dentate with light. The mice were then fear conditioned in A but tested in a different context B, with and without the dentate light stimulus on. As expected, the light stimulus did evoke fear reactions. In BACON direct activation of the DG' cells that were active during encoding of A would evoke a _CA3_Ptrn_Fnl_ that was a complete representation of context A even if only a portion of the relevant DG' cells were successfully activated. However, there would be a definite mismatch between the attributes of context B observed during the test and the recalled attributes of context A activated by the representation. This mismatch would degrade *B_Rep_* and decrease fear expression. Thus, contextual fear would be expressed when the context A representation was activated in context B, but consistent with the behavioral findings, the fear would be of sub-normal magnitude.

### Predictions, tested, and untested

BACON emulates many known aspects of real contextual fear conditioning (Results and Table 3), suggesting the possibility that biological context fear circuitry may work in a somewhat BACON-like way. It should be appreciated that the algorithm's ability to do this was not explicitly built in; it is a natural consequence of designing an algorithm that deals in an adaptive way with the problems posed by context fear learning. The algorithm also predicts a number of behavioral characteristics that are presently unknown (Table 3) and that seem worth experimental test. However, it should be emphasized that predictions that depend on the algorithm's Bayesian evaluations of context identity could well be falsified without it ruling out the more general idea that hippocampus operates in several distinctly different modes and that control of these is determined in some principled (but not necessarily Bayesian) way by comparisons between attributes of the current context and those associated with contexts from the animal's past.

### Unfinished business

The present work grew out of an earlier attempt to construct a fairly comprehensive model of fear conditioning that included cue as well as context conditioning (Krasne et al., 2011). That model accounted for much of the phenomenology of fear conditioning; however, hippocampal representations of context were assumed to develop gradually during contextual exposure, without any theory as to mechanism. The current algorithm supplies a mechanism, and ultimately the two models should be merged.

A number of simplifying assumptions were made during the design of BACON, and ways of moving beyond them should be explored in future work. Some matters especially needing consideration are these: (1) A theory of sampling. How does attribute sampling vary over time; does it slow as the set of attributes so-far observed during a session becomes more complete? (2). We assumed that all contexts have the same number of attributes. Ways of dealing with cases where this is an implausible assumption are needed. Weight of Evidence calculations depend on the total number of attributes available for sampling in a situation (our *N_A_*). How might this number be estimated on the fly without extensive prior sampling of a context? (3) It seems not unlikely that sampling slows or stops during freezing. If so, it would greatly affect BACON's behavior and needs to be considered as part of a theory of sampling. (4) Random sampling is a useful simplifying assumption, but some attributes are surely more salient than others. This ultimately should be incorporated into a model such as this. (5) BACON assumes that existing representations are activated serially and evaluated for validity as they become active; a more sophisticated algorithm that can compare validities of multiple attributes in parallel would produce better performance. (6) Extinction, which we chose to ignore, must be taken into account. It will be introduced into the alogrithm at a later time by assuming, as done in the above-mentioned model of the amygdala, that extinction occurs because unreinforced fear responses cause potentiation at synapses between active CA3′ neurons and amygdala interneurons that inhibit BACON's fear-producing neurons.

## Conclusion

We have constructed an algorithm, BACON, which randomly and serially samples attributes of its current context, uses its hippocampus to activate representations of whatever known context best matches the current one, and computes the Bayesian Weight of Evidence that the hippocampal representation is or is not that of the actual context. If this value indicates that the representation is valid, context fear conditioning and expression are enabled. If not, the hippocampus is configured to create a representation for the apparently novel context. This machinery works well, accounts for several well-known aspects of context fear learning, such as the immediate shock deficit, and makes new predictions. BACON's use of external control to enable hippocampal representation creation does away with several problems that have plagued thinking about hippocampal function (especially the conflict between pattern separation and completion and problems that arise if the “indirect” pathway from entorhinal cortrex to CA3 is used both during representation creation and recall). Overall, we believe that the approach taken provides insight into both context fear learning mechanisms and hippocampal function in general.

### Conflict of interest statement

The authors declare that the research was conducted in the absence of any commercial or financial relationships that could be construed as a potential conflict of interest.
