# Supplementary material for: A Bayesian context fear learning algorithm/automaton
Source: Front Behav Neurosci. 2015 May 27;9:112. doi: 10.3389/fnbeh.2015.00112 (PMC4445248; doi:10.3389/fnbeh.2015.00112)
Supplement: Supplementary file 1 [file Presentation1.PDF]

ON-LINE SUPPLEMENTARY MATERIALS

This document provides supplementary materials for **A Bayesian Context Fear Learning Algorithm/Automaton**, which we will refer to here simply as the "Paper". It goes into several issues that would either have taken too much space in the published paper or distracted too much from the main line of argument there. The matters considered are these:

A. Use of KWTA threshold setting in recurrent collateral auto-associator of CA3'
B. Conditions for autoassociator to activate a 'complete' representation
C. Exact calculation of Bayesian Weight of Evidence
D. A circuit that inhibits representation creation when there may already be a representation of the current context
E. ${}_{CA3}Ptrn_o$ when all attributes of two similar contexts have been encoded.
F. Simulation of Leutgeb et al (2007) two-room exp.
G. Pseudo-code for program flow.
H. Unspecified neural implementations.
I. References for this Supplement.

A. Use of KWTA threshold setting in recurrent collateral auto-associator of CA3'

Previous accounts of completely connected recurrent collateral networks, such as that of BACON's CA3' have made the threshold for firing of neurons equal to the number of active neurons providing input to the network at any given iteration (McNaughton and Morris, 1987). As a consequence, subsets of those neurons that were active during encoding would restore activity of the full original pattern, but any neuron not receiving effective input from all the active neurons would not fire, and spurious pattern completion would thereby be avoided. This method of setting auto-associator thresholds only works when input patterns at the time of recall are subsets of the encoded pattern. In the present usage, it is commonly the case that, whereas during recall in a given context there are *more* representations neurons of the current context than of other contexs active in ${}_{CA3}Ptrn_o$, there may nevertheless be quite a number of neurons active in ${}_{CA3}Ptrn_o$ that are part of the representation of contexts other than the current one.

Under such circumstances, the above threshold setting rule will work poorly at best. To see this, suppose that two similar patterns, A and B have been encoded. Each will be represented by some hippocampal neurons that are specific to it, while there will be some representation cells common to both patterns. Especially early in the process of sampling context attributes, there will be neurons specific to both A and B active in ${}_{CA3}Ptrn_o$ (see Figs 7 and 8 of the Paper). Suppose that there are K neurons active, of which N_A are specific to context A, N_B of which are specific to context B, and N_{AB} of which are part of the representation of both [$N_A+N_B+N_{AB}=K$; $N_A<K$ and $N_B<K$]. At the first iteration of recurrent collateral input the A-specific neurons will be effectively excited by N_A+N_{AB} inputs and the B-specific ones by N_B+N_{AB} inputs. Both sums will be $<K$. Only the representation cells that are part of both A and B will get K effective inputs and fire. So even though $N_A>N_B$, this information will be lost at the next iteration, and a complete representation of context A will never be able to be activated in CA3'.

B. Conditions for autoassociator to activate a 'complete' representation

As a preface to this section it will be useful to introduce some terminology: We will call a Hippocampal cell that represents only context x a "solo" context x cell, a cell that represents context x and also other contexts, a "mixed x" cell, and a cell that represents more than one context, without regard to which ones, simply a "mixed" representation cell. We will call the number of solo x cells active in ${}_{CA3}Ptrn_o$ S_x and the total number representing x (whether solo or not) T_x. Also call the total number of solo x cells that have been created L_x and the total number of mixed cells N_{mxd}.

As explained in the Paper, during recall it will usually be the case that the K ${}_{CA3}Ptrn_o$ cells activated by a sample of a context's attributes (call it context x) will include not only solo and mixed context x

representation cells but also cells that do not even represent context x. If sampling of a context is well advanced, eventually solo and mixed context x cells will be more common than cells representing any other context, but even then there will often be cells active that do not represent context x. As pointed out in the Paper, it is only after input from the recurrent collateral network that a 'complete' representation of context x comprised of the *K* cells of context x's representation and no others will be activated in $_{CA3}Ptrn_{fin}$. Thus arises the question of under what circumstances a complete representation will be produced at the output of CA3'? As we will explain below, three rules emerge from a consideration of this question (we call the current context x and assume that x is one of *M* similar contexts for which representations have previously been created):

Rule 1. If *M*=2, then a complete representation of context of x will be activated in CA3' as the result of one cycle of recurrent input if a majority of the $_{CA3}Ptrn_o$ cells represent x.

Rule 2. If *M*>2, then the condition for a complete representation at the first cycle of recurrent input is that $T_x > K - S_x$. This condition will be fulfilled only if *M* is quite low and the percent overslap (ω) of each of the *M* contexts is quite low. For example, if *M*=3, ω would have to be less than 70% (given that 50% of attributes in BACON are assumed to be common to all contexts. This means that a complete representation would occur after one cycle only if each pair of contexts had less than 30% of overlap of context-specific attributes). Thus, a complete representation of the current of more than 2 similar contexts would be unlikely to emerge from the first cycle unless the contexts were quite dissimilar.

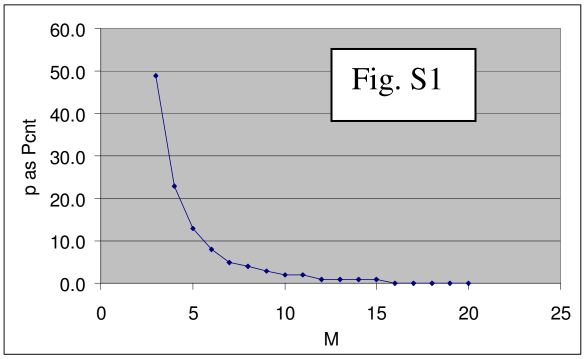

Rule 3. If a plurality of active cells in $_{CA3}Ptrn_o$ represent context x, then a complete representation will emerge at the end of the second cycle of recurrent input if $N_{mxd} < K$. If the probability that a unit that is part of one representation will also be part of a second is ρ, then

$$N_{mxd} = K \sum_{j=2}^{M} C(j, M)\, \rho^{j-1} (1-\rho)^{M-j} .$$

Where C( ) is the number of combinations of *M* things taken *j* at a time. Figure S1 plots the value of *ρ* for which $N_{mxd}=K$ when *K*=60, as was the case in the simulations of the Paper. Thus, if *M* were 5, ρ would have to be less than about 10% in order for a complete representation to become activated by two iterations of input from the recurrent collaterals.

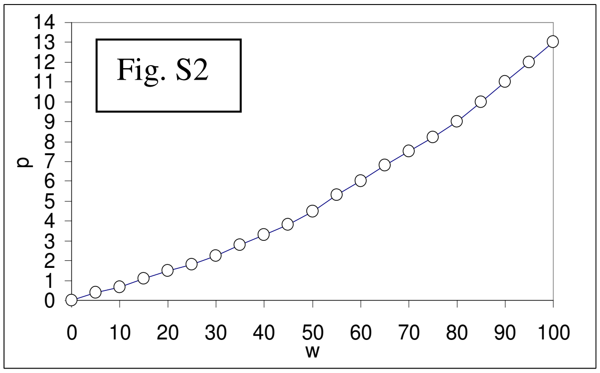

Fig. S2 shows the relationship between the probability of overlap between a given attribute in two different contexts (ω) and the probability of overlap of their hippocampal representations (ρ) when representations are formed at $Z_{cur}=Z_0$. Even when ω is very high, ρ is not more than about 13%. This is the case because two different samplings of Z_0 attributes, even of the same context, will end up with a somewhat different set of attributes, and the pattern separating features of DG' will decrease overlap still further. Since even for very similar (i.e. virtually identical) contexts ρ is less than 13%, one can usually expect to get a complete representation after two iterations if there are not more than 5 very similar contexts encoded.

Somewhat better performance might sometimes be achieved by allowing further cycles of recurrent input, but we believe that not much improvement can be achieved in that way, and we run just two cycles of recurrent input in BACON. Further explication of this matter is a topic for further investigation.

In order understand the reasons for the above rules, it is useful to first make several general points:

i). Each cycle of recurrent collateral input begins with a set of CA3'cells active. This activity then propagates into the cells' recurrent collaterals where it produces a unit of excitation (or "vote"). Excitation from the collateral-receiving dendrites is assumed to replace whatever excitation activated the cells and collaterals in the first place, and units of excitation are assumed to add (without decrement) proximally. The KWTA rule is then applied. A common threshold of the cells is set so that at least K fire. If there are ties, then more than K are activated (activation is all-or-none); this is in contrast to the situation for determinaton of the initial pattern of CA3' activity $_{CA3}Ptrn_o$ where any ties are broken randomly so that exactly K cells fire.

ii). During input from recurrent collaterals, each CA3' cell will get a unit of excitation (vote) from every cell that represents a context that it, itself represents.

Therefore a solo context x cell will get a unit of excitation from every solo or mixed CA3' cell that represents context x. Thus the excitation of a solo x cell is T_x.

A *mixed* cell that (in part) represents context x will *in addition* receive excitation from all those cells that do not represent context x but do represent other contexts which that mixed cell represents. Thus mixed cells representing a context will usually receive more excitation than their solo counterparts.

Explanation of Rule 1. If M=2, then all the mixed 1 and mixed 2 cells are the identical sets of cells. Therefore, if $T_1>T_2$, the excitation of the solo 1's will be greater than that of the solo 2s and the excitation of the mixed 1s will be greater than that of the solo 1s. That accounts for all the cells, so there will be no mixed cells other than mixed 1 cells having greater excitation than the solo 1s. The KWTA rule will thus cause the K context 1 cells and no others to fire.

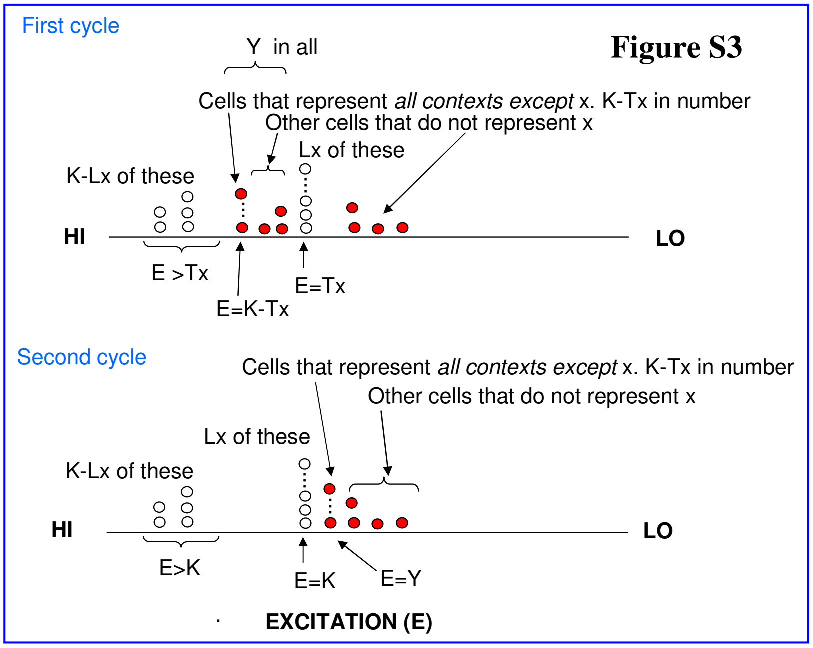

Figure S3

Explanation of Rule 2. As pointed out above, mixed cells representing a context receive more excitation than their solo counterparts. So when the KWTA rule is applied, one will get a complete representation of context x if all non-x representation cells have excitation less than that of the solo x cells, which is T_x. The non-x cells with the greatest excitation will be those which represent all contexts but x, and they will be excited by all active cells but the solo x cells, of which there are K-S_x. Therefore, the criterion for a complete representation at the end of the first cycle of recurrent input is $T_x>K-S_x$ (or $S_x>K-T_x$). The calculations to determine expected values of T_x and S_x for given M and ω are complex, and since they show that only quite distinct contexts will be able to meet the above criterion, we do not detail the calculations here.

Explanation of Rule 3. If T_x is greater than all other T_js, then the solo x cells will be more excited than any other type of solo cell, and the excitation of the mixed x cells will be greater still. If the total number of mixed cells < K, then there will be less than K mixed cells of any kind having excitation greater than that of the solo x cells.

Since the total number of mixed cells is <K, the total number of cells firing at the end of the first pass will be the K context x rep cells (white in Fig. S3) plus Y (<K) non-context x cells (red).

At the second pass the (K-L_x) mixed context x cells will be excited by the K context x cells plus some of the non context x cells, and the solo x cells will be excited by the K context x cells active at the end of

the previous pass. The most excited non-context x cells will be the set representing every context except x, and they will be excited by the $Y<K$ active non-context x cells. So all non-context x cells will get less excitation than any of the context x cells. Hence there will be a 'complete' representation of context x.

C. Exact calculation of Bayesian Weight of Evidence

Given the values of Z_{com}, Z_{cur}, and Z_{rec} we define the Bayesian Weight of Evidence that an active representation is valid as

$$B_{Rep}(Z_{com} | Z_{cur}, Z_{rec}) = \log [P(Z_{com} | Same, Z_{cur}, Z_{rec}) / P(Z_{com} | Diff, Z_{cur}, Z_{rec})$$

where *Same* specifies that the active representation is valid and *Diff* that it is not and that instead the automaton is in some random context. Here, and in the equations below, "Z_{cur}" means that the variable Z_{cur} has the value Z_{cur}, etc.

It is convenient to define some additional variables: Z_v, Z_g, Z_r
Z_r~Number of attributes of the current context (whether sampled or not) that are asssociated with the currently active hippocampal representation.
Z_g~Number of general attributes (i.e. attributes that pertain to all contexts) that are associated with the currently active hippocampal representation
Z_v~Number of context-specific attributes of the current context that are associated with the currently active hippocampal representation.

The *Same* case is straight-forward:
$$P(Z_{com} | Same, Z_{cur}, Z_{rec}) = H(Z_{com} | Z_{cur}, Z_{rec}, N_A)$$

The *Diff* case is more complicated: Note that every context has all the N_g general attributes but only N_A-N_g of the N_{ctx}-N_g possible context-specific ones.

$$P(Z_{com} | Diff, Z_{cur}, Z_{rec}) = \sum_{Z_r=j}^{N_A} H(Z_{com} | Z_{cur}, j, N_A)\, P(Z_r)$$

where

$$P(Z_{com} | Diff, Z_{cur}, Z_{rec}) = \sum_{Zr=k}^{N_A} H(Z_{com} | Z_{cur}, j, N_A)\, P(Z_r=k)$$

and

$$P(Z_r=k) = \sum_{\substack{\text{All } Z_v=i \text{ and } Z_g=j \\ \text{such that } i+j=k}} P(Z_v=i \text{ and } Z_g=j) = \sum_{\substack{\text{All } Z_v=i \text{ and } Z_g=j \\ \text{such that } i+j=k}} H(i|N_A-N_g, Z_x - j, N_p)\, H(j|Z_x, N_g, N_A)$$

D. Circuit that inhibits representation creation when there may already be a representation of the current context

At each computational cycle the basic algorithm that constitutes BACON activates whatever existing contextual representation receives the most input from those contextual attributes that have so far been randomly sampled during the current session. It then computes the Bayesian weight of evidence (B_{Rep}) that this representation is valid. If B_{Rep} is less than B_{new} the active representation is almost certain not to be valid. The active representation will be the existing representation most consistent with the set of

attributes so far sampled. So if sampling is far advanced and the "best available" representation is definitely invalid, the appropriate action is to infer that the automaton is somewhere for which there is no existing representation and create a new one for this context. And this is what the neural circuit presented in the paper proper and shown at the top of Fig. S4 would do. However, if sampling in a given session is not far advanced the viscissitudes of attribute sampling my well cause an invalid representation to be activated. In such cases a negative B_{Rep} value will be calculated, but usually it will not be very negative because Z_{cur} is small, and the automaton will go on to further sampling of the current context without alterning its set of representations in any way. However, if it should happen that the Z_{rec} value associated with the active representation is very high (i.e. a lot is known about the invalid context whose representation is active), the circuit shown at the top of the figure would create a new representation if $B_{Rep} < B_{new}$. However, this might well be a mistake, because a valid representation for this context might already exist (but not be active because of the accidents of current sampling).

As an add-on to try to diminish such occurrences, we added to the rules for creating a new representation the proviso that, even though the B_{Rep} value for an active representation is less than B_{new}, a new representation should not be created if at some prior point in the session a representation with a fairly high B_{Rep} value (taken to be a value greater than B_{tv}) had been activated by the set of attributes sampled. This is not an absolute fix, but it dramatically decreases unwanted creations of second representations for contexts already having hippocampal representations. Presentation of circuitry that could implement this rule was deferred to this supplement, and is shown at the bottom-right of Fig. S4

The added circuitry, prevents neuron C from activating the Create neuron if there is any representation the automaton has earlier in the session evaluated as tentatively valid but not subsequently rejected: Whenever a representation producing a $B_{Rep} > B_{tv}$ is activated, the synapses of all active Hipp II neurons on neuron F (those orange synapses that are active) become potentiated. Unless this potentiation is reversed, whenever that representation is active, it will activate the F neuron and prevent the Create neuron from firing. This potentiation can only be undone if the representation is activated in conjunction with the C neuron, which itself signals $B_{Rep} < B_{new}$. Thus the Create neuron cannot be activated if there are any representations that have been judged tentatively valid but not subsequently been rejected.

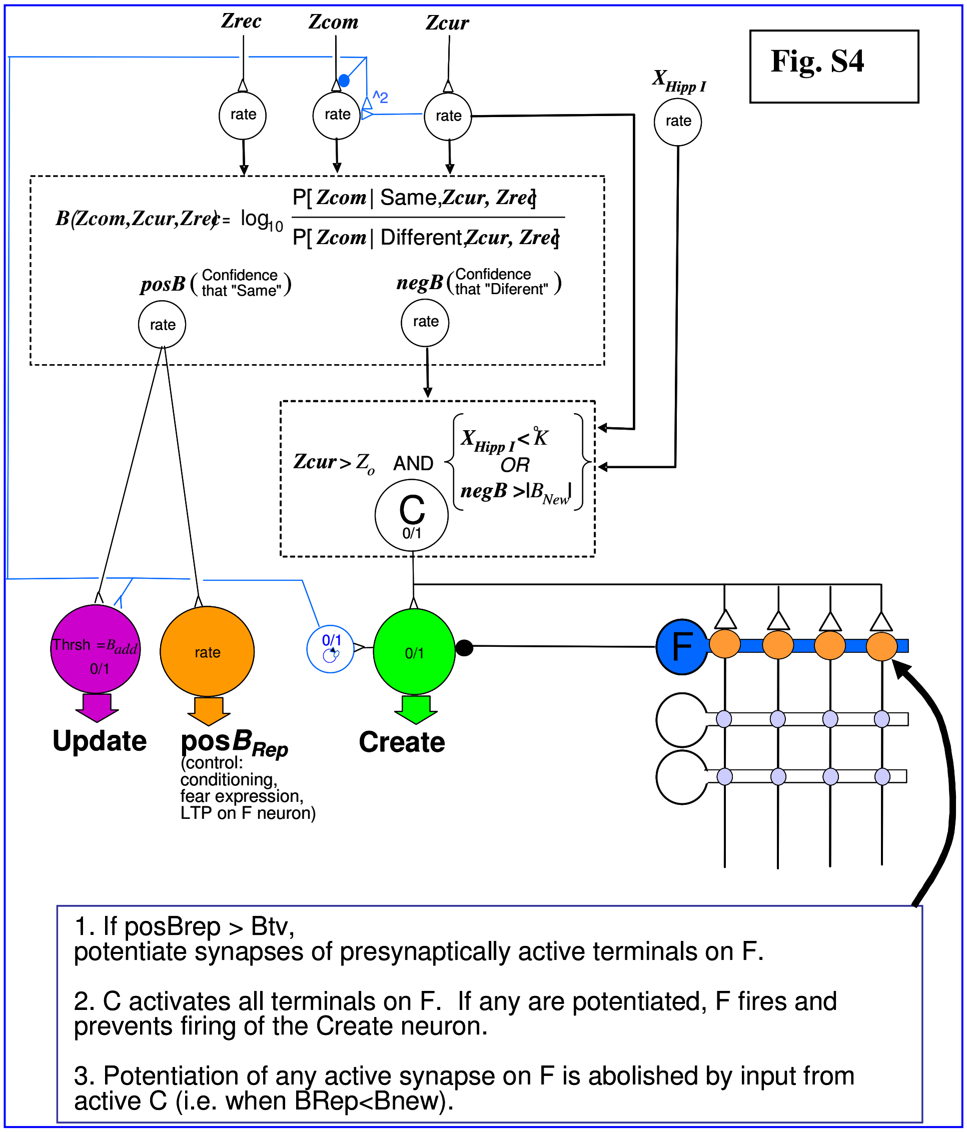

E. $_{CA3}$Ptrn$_o$ when all attributes of two similar contexts have been encoded.

If several similar contexts are known, $_{CA3}$Ptrn$_o$ will usually include representation cells specific to contexts that are similar to those it is in as well as cells specific to the actual context, though cells representing the actual context will predominate. The "errors" will occur in part because partial random sampling of attributes during encoding and updating causes different common attributes to become associated with each representation, and attributes associated with a "wrong" representation may be sampled during recall. But even if all N_A attributes of two similar contexts have been encoded, representation cells specific to the wrong one may be among the K most excited because many of the common attributes so far sampled happen to be ones that innervate those particular cells. The simulation results shown at the right illustrate this point.

Fig. S5

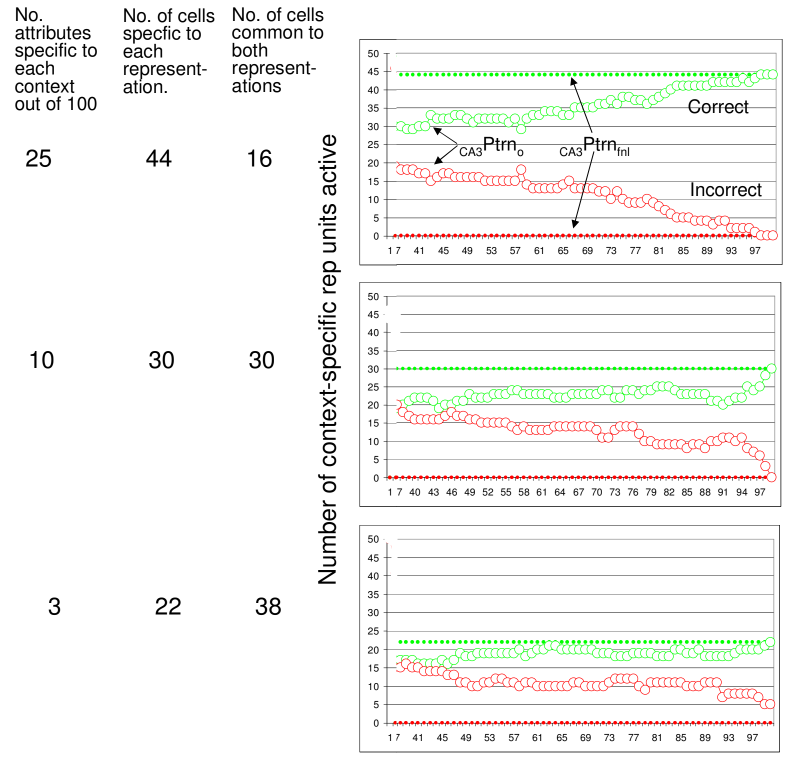

Two contexts having the number of context-specific attributes indicated in the left-most column were fully encoded, and BACON then tested for recall in one of them. The complete representations of the two contexts overlapped to the extent indicated in the 2nd and 3rd columns of the figure. When 25% of the contexts' attributes were non-overlapping, 73% of their representation cells were distinct. When only 3% of their attributes were overlapping, 37% of their representation cells were distinct.

The graphs at the right show how many of the distinct representation cells for the context in which BACON was placed, and also for the other context, were active as more and more of the context's 100 attributes were sampled (the graphs start at sample 17). At all the stages of sampling shown, $_{CA3}$Ptrn$_{fnl}$ was the complete representation of the context BACON was actually in. However in $_{CA3}$Ptrn$_o$ there were representation cells specific to the incorrect context active throughout most of the sampling process, though these were less numerous than those of the correct representation. It was only after sampling was very advanced that incorrect cells stopped being part of the active set, and for the most similar pair of contexts, some continued to fire even after all 100 attributes had been sampled.

F. Simulation of Leutgeb et al (2007) two-room exp.

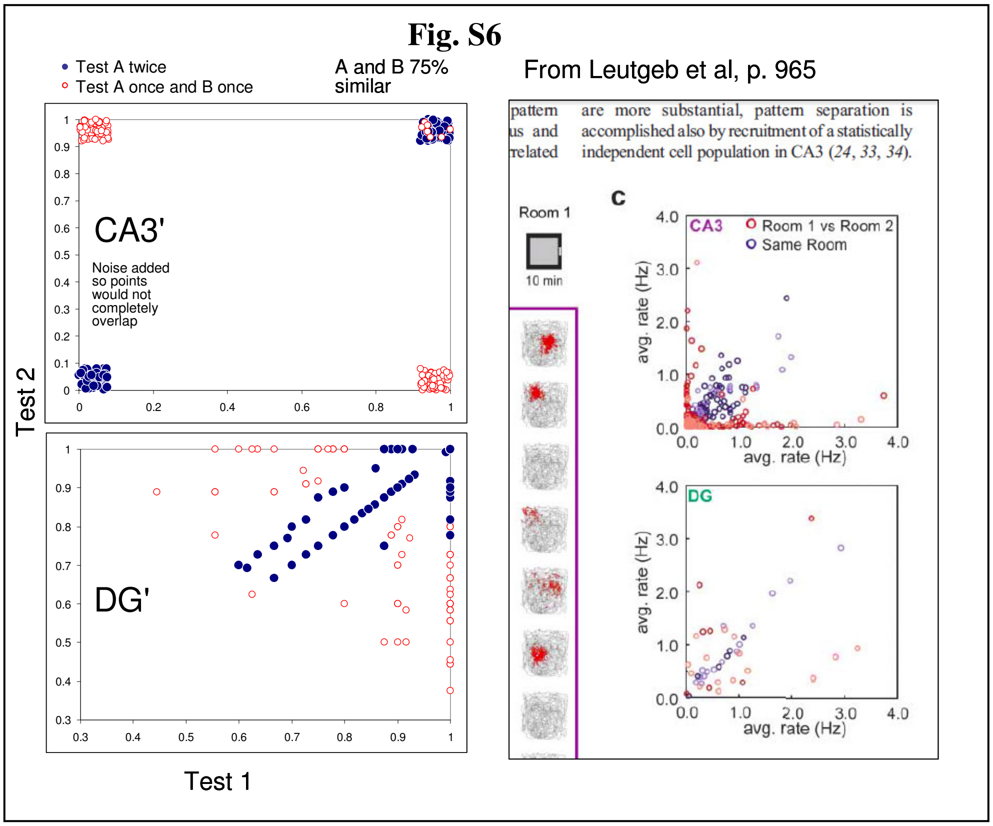

At the right of Fig. S6 is a page from an experiment by Leutgeb et al (2007), which was discussed in the Paper proper. Rats were familiarized with two contexts, A and B and then tested several times in each while recording from both dentate and CA3. The red points on each scatterplot show the firing rate of a given cell in both context A (one axis) and context B (the other). The dark blue points are for two different sessions in the *same* context. In CA3, when tested in two different contexts most cells responded in only one of them, whereas in DG they tended to respond in both, but at different rates in each. When tested twice in the same context, both the CA3 and the DG cells responded at about the same rate on each occasion in the context.

The graphs at the left of the figure show simulation results for a similar experiment in BACON. The automaton was given an encoding session of 80 samples each in context A and context B, which had 75% of their attributes in common (50% of their non-general attributes). It was then tested either twice in A or once each in A and B. The results shown are for the Z_{cur}=95 in each session. As in the real animals, CA3 cells responded similarly on each test in context A when tested in the two different contexts, most responded in only one (except for the 7 of 113 total representation cells that were common to both contexts). These results are similar to those of the real animals except that when a BACON cell did respond, it always responded at a fixed high rate whereas in the real animals different cells responded at different rates. It may well be that the firing rate of CA3 cells in real animals are coding for something not mimicked by BACON. However, as pointed out in our paper, one way of coding B_{Rep} would be via the firing rate of representation cells. If that were happening in real animals, then B_{Rep} values would be different in each of the several animals from which Leutgeb et al's data were obtained, and a range of firing rates might well have been seen. In DG, which in BACON does use rate coding during recall, the results are even more similar to those of the real animals. The most obvious difference is that there are no very low firing rates in the BACON simulation. This is because all cells that became representation cells did so because they were fairly well innervated by the contexts encoded, and since 50% of attributes are common to all contexts (N_{Gen}=50), most cells that got encoded got some input during the test (Z_{cur}=95). Had we included data from earlier in the session, there would have been more low firing rates in the scatterplot.

The Leutgeb et al paper found that the proportion of sampled cells firing in DG was about a third that in CA3'. As discussed in the BACON paper, we could have achieved greater biological realism by letting BACON's CA3' be about an eighth smaller than DG', with no impact on the performance of the automaton unless large numbers of very similar contexts had to be learned. If we had made this size adjustment, then the numbers of cells active during tests in the above experiment would have been as shown at the right. In CA3', which is at all times subject to a KWTA rule, exactly *K* cells would have fired, leading to the firing rate distributions shown at the top of Fig. S7. In DG' there is a range of firing rates during recall. If only one context has been learned, then the picture is like that at the left of the figure, where the total *number* of cells firing is *K* (=60). If two contexts have been learned, the picture depends on how similar the contexts are to each other If they are very different (as on the far right where only the 50 attributes common to all contexts are the same in A and B), there is a group of cells that fire in the same way as if only one context were encoded and another that fire at lower rates. If they are very similar, as in the middle column of graphs, the number of cells active is 2*K* - the number of representation cells common to the two contexts, and there is a spread a little greater than in the left-most case.

Fig. S7

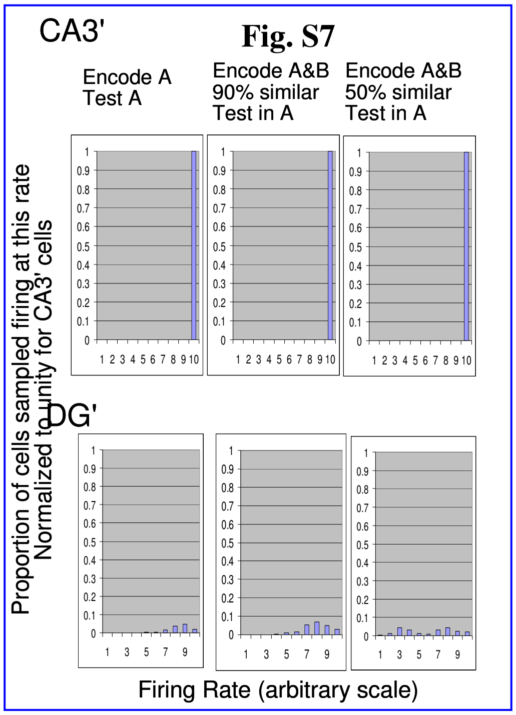

If a great many very different contexts have been encoded, there will be on the order of *K* representation cells created for context, and thus the total number of cells active at recall could become quite large. However, the representation cells for the correct context would fire at much higher rates than those of incorrect ones. Moreover, since the CA3’ partners of relatively unexcited DG’ cells will never be winners of a CA3’ KWTA competition, our algorithm could have assumed a regulatory mechanism that silenced DG' cells excited so little that they could have no impact on CA3' behavior. Had we done that, the number of DG' cells firing would remain modestly greater than *K* no matter how many contexts were encoded.

G. Pseudo-code for computation cycle

We give here the flow diagram of computer program that implements BACON.

Subroutines and functions are in bold-face; variables or flags are ordinary font, and logical statements are in caps. The role of Subroutine and Function names are mostly obvious in the context of the paper.

Sample makes a random selection of a not-yet-sampled contextual attribute and augments Z_{cur} by one. **EvaluateHipp** calculates excitations and graded firing rates of DG' cells, calculates excitation of CA3' cells and determines $_{CA3}Ptrn_o$, and $_{CA3}Ptrn_{Fnl}$. **EvaluateCtx** causes the firing those EC_{out} cells for which more than *ThrshCtx* of its active inputs have potentiated synapses with the cell (EC' cells are binary). It also computes Z_{rec} and Z_{com}. **MakeNewRep** determines which DG'-CA3' dyads have heaviest innervation from EC'_{in} and then potentiates all appropriate EC'in-DG', EC'in-CA3', CA3'-CA3', and CA3'-EC'out synapses, normalizing those made by EC'_{in}. **Update** potentiates input and output synapses between the currently active representation cells and EC'_{in} and EC'_{out} neurons that represent all current or recalled attributes, normalizing those made by EC'_{in}.

Sample
EvaluateHipp
EvaluateCtx
IF NewRep=0
  EvaluateBrep
ElSEIF NewRep=1
  EvaluateExpectedBrep
  Brep=ExpectedBrep
END IF
EvaluateFear(Brep)
IF Brep<=Bnew AND NewRep=0
  NewRep=1
  Add2Rep=1
  MakeNewRep
ELSEIF Brep>=Badd
  Add2Rep=1
  IfFAdd2Rep=1 OR NewRep=1, **Update**
ELSE
  Add2Rep=0
  IF Add2Rep=1 OR NewRep=1, **Update**
END IF
EvaluateHipp
EvaluateCtx
IF NewRep=0
  EvaluateBrep
ELSEIF NewRep=1
  EvaluateExpectedBrep
  Brep=ExpectedBrep
END IF
EvaluateCnd(Brep)
IF Shk=1, **DoShk(Cnd)**

H. Unspecified neural implementations

There are several places in this paper where neural computations are simply assumed to occur, without providing a means of neural implementation. This was done for K-winners-takeall logic because this is a very non-straightforward topic that has been discussed elsewhere (e.g. Coultrip et al., 1992;Wang, 2010;Tymoshchuk, 2013) and would have distracted here. Neural circuitry to carry out the logic in the box feeding the Create neuron in **Figure 7** of the Paper, which could be implemented in many ways, was also not specified. We freely used neurons which fire at rates proportional to sums, differences, products, and power functions of the firing rates of the neurons innervating them. Sums are straightforward so long as synaptic conductances are kept small relative to leakage conductances of neurons. Subtraction can be accomplished by inhibition, though distortion by shunting effects of inhibition must be contended with; methods that do not have this problem have been suggested (Holt and Koch, 1997). Products could be computed using divisive inhibition to divide by the reciprocal of a multiplier. Several additional ways of computing products have been suggested (e.g. Tal and Schwartz, 1997;Gabbiani et al, 2002;Nezis and vanRossum, 2011), and there is empirical evidence that, whatever the biophysical mechanism responsible, actual neurons can do so (Silver, 2010; Gabbianai et al, 2002). Outputs that are accelerating functions of inputs, and therefore power-function-like, could be produced by allowing limited active responses to membrane depolarization, such as those that can be produced by voltage-dependent calcium channels in dendritic membrane.

I. References for this supplement

Coultrip, R., Granger, R., and G, L. (1992). A cortical model of winner-take-all competition via lateral inhibition. *Neural Networks* 5**,** 47-54.

Gabbiani F, Krapp HG, Koch C, Laurent G (2002). Multiplicative computation in a visual neuron sensitive to looming. *Nature* 420, 320-4.

Holt, G.R., and Koch, C. (1997). Shunting inhibition does not have a divisive effect on firing rates. *Neural Computation* 9**,** 1001-1013.

Leutgeb JK, Leutgeb S, Moser MB, Moser EI. (2007). Pattern separation in the dentate gyrus and CA3 of the hippocampus. *Science*.315(5814):961-6.

Nezis P, vanRossum MC (2011). Accurate multiplication with noisy spiking neurons. *J Neural Eng* 8,(3):034005. doi:10.1088/1741-2560/8/3/034005.

Silver, R.A. (2010). Neuronal arithmetic. *Nature Reviews Neuroscience* 11**,** 474-489.

Skaggs, W.E., and Mcnaughton, B.L. (1992). Computational approaches to hippocampal function. *Curr Opin Neurobiol* 2**,** 209-211.

Tal, D., and Schwartz, E.L. (1997). Computing with leaky integrate-and-fire neuron: logarithmic computation and multiplication. *Neural Computation* 9**,** 305-318.

Tymoshchuk, P.V. (2013). A model of analogue K-winners-take-all neural circuit. *Neural Netw.* 42**,** 44-61.

Wang, J. (2010). Analysis and design of a k-winners-take-all model with a single state variable and the Heaviside step activation function. *IEEE Transactions on Neural Networks* 21**,** 1496-1506.
